# Subspace-constrained approaches to low-rank fMRI acceleration

**DOI:** 10.1016/j.neuroimage.2021.118235

**Published:** 2021-06-03

**Authors:** Harry T. Mason, Nadine N. Graedel, Karla L. Miller, Mark Chiew

**Affiliations:** a Wellcome Centre for Integrative Neuroscience, FMRIB Centre, University of Oxford, Oxford, United Kingdom; b Wellcome Centre for Human Neuroimaging, UCL Institute of Neurology, London, United Kingdom

**Keywords:** fMRI, Acceleration, Temporal Resolution, Low Rank, k-t FASTER, Tikhonov Regularization, Temporal Smoothing, Low Resolution Priors

## Abstract

Acceleration methods in fMRI aim to reconstruct high fidelity images from under-sampled k-space, allowing fMRI datasets to achieve higher temporal resolution, reduced physiological noise aliasing, and increased statistical degrees of freedom. While low levels of acceleration are typically part of standard fMRI protocols through parallel imaging, there exists the potential for approaches that allow much greater acceleration. One such existing approach is k-t FASTER, which exploits the inherent low-rank nature of fMRI. In this paper, we present a reformulated version of k-t FASTER which includes additional L2 constraints within a low-rank framework.

We evaluated the effect of three different constraints against existing low-rank approaches to fMRI reconstruction: Tikhonov constraints, low-resolution priors, and temporal subspace smoothness. The different approaches are separately tested for robustness to under-sampling and thermal noise levels, in both retrospectively and prospectively-undersampled finger-tapping task fMRI data. Reconstruction quality is evaluated by accurate reconstruction of low-rank subspaces and activation maps.

The use of L2 constraints was found to achieve consistently improved results, producing high fidelity reconstructions of statistical parameter maps at higher acceleration factors and lower SNR values than existing methods, but at a cost of longer computation time. In particular, the Tikhonov constraint proved very robust across all tested datasets, and the temporal subspace smoothness constraint provided the best reconstruction scores in the prospectively-undersampled dataset. These results demonstrate that regularized low-rank reconstruction of fMRI data can recover functional information at high acceleration factors without the use of any model-based spatial constraints.

## 1 Introduction

fMRI is a non-invasive, whole-brain functional imaging technique that suffers from a trade-off between temporal and spatial resolution. Acceleration aims to increase the temporal resolution without loss of spatial resolution through higher sampling efficiency in conjunction with advanced image reconstruction that leverages additional information and/or constraints. By providing increased temporal degrees of freedom in a given scan duration, acceleration can: improve sensitivity to temporal features of the haemodynamic response; reduce physiological noise aliasing; and improve statistical power. Depending on the application, the increased sampling efficiency garnered from acceleration could also be used to reduce scan times, or to increase the spatial resolution.

Various acceleration techniques have been widely adopted for fMRI. Parallel imaging methods rely on the spatial variation of sensitivity profiles of multi-channel receiver coils, which provide additional spatial information in image reconstruction. This can occur in the image domain (e.g. SENSE Pruessmann et al., 1999) or in the sampling domain (e.g. GRAPPA Griswold et al., 2002). Simultaneous multi-slice imaging (Setsompop et al., 2012; Barth et al., 2016) extends these in-plane techniques to accelerate across slices without significant reduction factor SNR penalties when compared to 2D methods, since the under-sampling can be offset by acquiring more slices (even accounting for the loss in SNR due to Ernst angle and shorter TR), increasing the achievable temporal resolution. Parallel imaging is conventionally a timepoint-by-timepoint approach that does not leverage any temporal information during reconstruction.

Methods that do jointly consider k-space and time are known as k-t methods and can be broadly separated into three categories: methods that make a strong assumption about the spatiotemporal structure (Madore et al., 1999; Tsao et al., 2003; Huang et al., 2005; Yun et al., 2013), methods that make a strong assumption about sparsity within a pre-defined basis set (compressed sensing approaches) (Lustig et al., 2007; Holland et al., 2013; Jeromin et al., 2012; Zong et al., 2014; Chavarrías et al., 2015), and methods that assume the data fits a globally low-rank model (Liang, 2007; Chiew et al., 2015). There are also approaches which combine these methods (Chavarrías et al., 2015; Pedersen et al., 2009; Jung et al., 2009; Qin et al., 2019; Otazo et al., 2015; Petrov et al., 2017). By focusing on redundancies or structural features in k-t space, k-t methods have the potential for much greater degrees of acceleration than time-independent methods due to the extra dimension of shared information.

Compressed sensing approaches use L1-constraint methods to promote sparsity in reconstruction. These approaches have proven very effective in other fields of dynamic MRI reconstruction, but have had relatively limited adoption in fMRI, likely due to difficulty finding suitable sparse representations for the relatively subtle BOLD signals. While initial exploratory work in compressed sensing reconstruction for fMRI focused on spatial-domain sparse transformations (Holland et al., 2013; Jeromin et al., 2012), most recent work incorporating sparsity assumptions have focused instead on sparsifying the temporal domain (Aggarwal and Gupta, 2017; Fang et al., 2016). Low rank + Sparse (*L* + *S*) methods (Otazo et al., 2015; Petrov et al., 2017), are a recent set of combined approaches that aim to isolate the functional information in the sparse component of the reconstruction (Singh et al., 2015; Aggarwal et al., 2017) while capturing the non-sparse background in the low-rank component. The result of this approach is that the rank in the L component is kept very low and that the majority of the important BOLD information is in the S component, with PEAR (Weizman et al., 2017) a notable recent example that explored the idea of capturing more BOLD information in the L component.

An alternative to sparse modelling of the BOLD signals is a conceptually simpler approach based on a regularized globally low-rank model of the fMRI data. There is a correspondence between the approaches that use training data to estimate a sparse or low-dimensional basis (Chavarrías et al., 2015; Jung et al., 2007) and low-rank models, since low-rank models by definition have few non-trivial components (i.e. the singular value distribution is sparse). However, low-rank models do not require prior knowledge of the sparse bases, and instead estimate the spatio-temporal basis representations for the data. The inherent low-rank nature of fMRI (Chiew et al., 2015), which can be understood as the combination of a few spatially coherent temporal processes (i.e. activation maps that identify voxels with a common time-series), forms one such exploitable structure in a k-t representation of the data. In analysis of fMRI data, for example, a dimensionality reduction is often applied as a pre-processing step (McKeown et al., 1998), which explicitly enforces a low-rank representation of the system prior to resting-state analysis methods such as independent component analysis (ICA) (Hyvärinen, 1999; Beckmann and Smith, 2004; Kiviniemi et al., 2003). Various noise sources (e.g. thermal noise, physiological noise, etc.), motion, and image artefacts make the system only approximately low rank, although some confounds can also be estimated as low-rank processes (Salimi-khorshidi et al., 2015).

Globally, low-rank methods can be used to represent space-time data as a spatial subspace paired with a temporal subspace and associated weighting factors. The Partially Separable Functions method (k-t PSF) (Liang (2007); Lam et al., 2013) is a data-driven approach that first identifies a temporal subspace from fully-sampled low spatial resolution and high temporal resolution training data, and then uses this to reconstruct a high resolution spatial subspace from under-sampled data. An alternative rank-constrained approach is k-t FASTER (fMRI Accelerated in Space-Time via Truncation of Effective Rank (Chiew et al., 2015; Chiew et al., 2016), which jointly identifies the subspaces that best describe the acquired data. Importantly, the only constraint imposed by k-t FASTER is that of fixed rank. The rank constraint alone is enough to achieve modest acceleration factors (Chiew et al., 2015), but rank-constrained methods may also be combined with coil sensitivity information and non-Cartesian sampling (Chiew et al., 2016) for increased acceleration.

In addition to the rank and coil sensitivity constraints, other information may also be incorporated into the reconstruction. Tikhonov regularization prevents overfitting on the temporal and spatial components, and serves as a way to penalize the energy content of the reconstruction. Radial k-space trajectories have a higher sampling density in central k-space than peripheral k-space, and so reweighting the low-resolution k-space could allow the reconstruction to be more strongly constrained in the densely sampled centre of k-space. The importance of central k-space more generally in MRI reconstruction has previously been used in approaches such as keyhole (Yun et al., 2013), k-t SPARSE (Lustig et al., 2007), and k-t PCA (Pedersen et al., 2009). Temporal regularization of some form has previously been incorporated into fMRI reconstruction in approaches like Dual TRACER (Li et al., 2018) and temporal smoothness for simultaneous multi-slice EPI (Chiew and Miller, Dec. 2019), with the latter specifically demonstrating smoothness producing a net improvement in tSNR and GLM efficiency. With a temporally varying sampling scheme, such as golden angle radial sampling (e.g. TURBINE Graedel et al., 2017), enforcing temporal smoothness can be an effective way to reduce aliasing artefacts with a fractional penalty to the resulting temporal degrees of freedom.

In this paper, we explore extensions to the k-t FASTER approach that are formulated within an alternating minimization framework that incorporates L2-based regularization in tandem with the previously established fixed-rank constraints. We explore specific L2 constraints that correspond to Tikhonov regularization, low-resolution priors, and temporal subspace smoothness. Using L2-based constraints allows for interpretations of the constraints as Gaussian priors, as they are robust and relatively simple to implement. We compare the proposed approaches to unconstrained k-t FASTER and k-t PSF reconstructions of retrospectively and prospectively under-sampled datasets, which can be conceived of as special cases within this regularization framework. We evaluate these different methods with regards to the accuracy of the spatial and temporal components, and the sensitivity and specificity of statistical parameter maps (activation).

## 2 Material and methods

### 2.1 Theory

#### 2.1.1 Reformulation of k-t FASTER

The original k-t FASTER methodology used an iterative hard threshold + matrix shrinking approach (Chiew et al., 2015) to enforce a fixed low-rank constraint on the reconstructed image time series. To enable us to easily introduce additional constraints on the spatial and temporal subspaces, we reformulate the low-rank k-t FASTER optimization as a matrix factorization problem. This alternate k-t FASTER formulation is equivalent to the original k-t FASTER formulation (Mason (2020)), with the main difference being the manner in which the rank constraint is enforced.

(1) $$\begin{array}{l} X,T=argmin_{X,T}(∥\text{E}(\text{X}*{\text{T}}^{\prime })-\text{d}{∥}_{2}^{2})\\ \;\text{such}\;\text{that}\;:\;\text{rank}(X)=rank(T)=r \end{array}$$

Eq. (1) uses the following variables - E: sampling and multi-coil encoding function; d: multi-coil under-sampled k-t fMRI data; X: spatial components of decomposition; T: temporal components (T’= Hermitian adjoint of T); ∥ ∥_2_ : L2 norm, and r: rank constraint. For non-Cartesian sampling, E will contain an NUFFT operator (Fessler and Sutton, 2003).

The rank constraint is implicitly enforced in our formulation through the shape of X and T, and so will be omitted from Eqs. (2)–(5) for brevity.

To solve the non-convex low-rank reconstruction, a minimization approach is used which alternately optimizes two convex subproblems (Jain et al., 2013). These subproblems solve for either the spatial (X) or temporal (T) components, respectively, while the other variable is fixed. The spatial dimensions are vectorized, such that the product X*T’ forms a 2D space-time low-rank matrix that is our estimate of the fMRI time-series, and the 3D image volumes are a re-formatting of the 1D spatial vector. The decomposed matrices X and T form a low-rank decomposition, with the low-rank structure encoded in the dimensionality of the matrices. X and T are not necessarily forced to be orthogonal, although orthogonalization was found to speed up reconstruction where no additional constraints were present (i.e. k-t FASTER and the prior generation), with no significant change in reconstruction output recorded. Pseudocode is included in Appendix A, and full implementation details are included in Appendix B.

#### 2.1.2 Soft constrained-subspace approaches

**Fig. 1:**
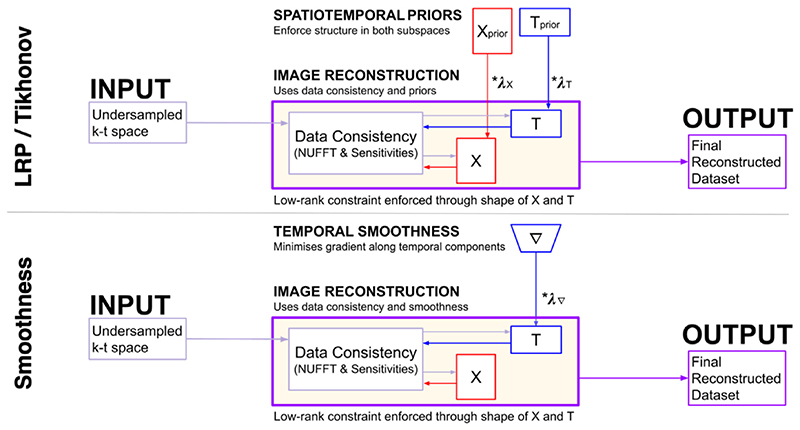
A schematic overview of a reconstruction via various constrained-subspace approaches. For the LRP, X_prior_ and T_prior_ are created using a windowed version of the under-sampled data according to only the rank constraints and coil sensitivity information. For Tikhonov, X_prior_ and T_prior_ are zero-filled. X_prior_ and T_prior_ are fed as a constraint into the final reconstruction, combining with the data consistency term on an unwindowed dataset to produce the final output. The temporal subspace smoothness schematic shows a finite difference matrix ∇ applied solely to the temporal component matrix T, before also being combined with the data consistency term.

The alternating minimization approach allows us to easily add additional subspace-specific constraints into Eq. (1), allowing us to enforce L2 constraints concurrently with the low-rank constraint. The relative influence of any L2 constraints within a reconstruction are controlled by regularization parameters (*λ*). The original k-t FASTER approach (Eq. (1)) can be derived by setting *λ* = 0 in all the following equations. Formulations with non-zero and non-infinity *λ* will be referred to as softly constrained. Fig. 1 contains schematics that demonstrate the various approaches.

##### Tikhonov

The most straightforward constrained-subspace approach derives from methods used for collaborative filtering (Koren (2009)), which often uses Tikhonov regularization on both component matrices (X and T). L2-regularization terms are included to serve as energy minimization terms for each variable, which prevent matrix entries from becoming too large:

(2) $$X,T=argmin_{X,T}(∥\text{E}(\text{X}*\text{T})-\text{d}{∥}_{2}^{2}+\lambda_{\text{X}}∥\text{X}{∥}_{2}^{2}+\lambda_{\text{T}}∥\text{T}{∥}_{2}^{2})$$

##### Low-Resolution Priors

For data acquired using trajectories with non-uniform sampling densities that sample the centre of k-space each TR, one can formulate an L2 regularization corresponding to Low-Resolution Priors (LRP). In uniform radial sampling drawn from multiple spokes (TRs) within a plane, a central window of radius $\frac{k_{-}\text{max}}{R}$ fulfils the Nyquist sampling criteria in the azimuthal direction. Additionally, these low spatial frequencies represent the net balance of temporal processes at the ultimate temporal resolution, but without capturing detailed spatial features. This central window can be more strongly weighted during a final reconstruction to accurately capture these high temporal resolution processes.

The LRP constraints (X_prior_ and T_prior_) are created by windowing the full k-space dataset with a Tukey window $\left(\text{FWHM}:\frac{\pi *k_{-}\text{max}}{2R}\right)$ and then reconstructing X and T using Eq. (1), albeit with *d* referring to windowed k-space data, analogous to the estimation of the temporal subspace from training data in the k-t PSF approach. The final reconstruction is then weighted by the LRPs along with the full unwindowed sampled data (Eq. (3)).

(3) $$X,T=argmin_{X,T}(∥\text{E}(\text{X}*\text{T})-\text{d}{∥}_{2}^{2}+\lambda_{\text{X}}∥\text{X}-{\text{X}}_{\text{prior}}{∥}_{2}^{2}+\lambda_{\text{T}}∥\text{T}-{\text{T}}_{\text{prior}}{∥}_{2}^{2})$$

The previously proposed k-t PSF method represents a special case of the more general LRP framework. This method reconstructs the spatial coefficients against a temporal basis (or prior) estimated from low-resolution training data. k-t PSF can be formulated in the Eq. (3) framework by setting *λ_X_* = 0 and *λ_T_*= ∞. The temporal subspace is constrained to be identical to this predetermined basis, which is labelled T_prior_:

(4) $$\begin{array}{l} X=argmin_{X}(∥E(\text{X}*\text{T})-\text{d}{∥}_{2}^{2});\\ T=T_{\text{prior}} \end{array}$$

##### Temporal Subspace Smoothness

A temporal subspace smoothness term aims to preserve the relatively smooth BOLD response (particularly at high acceleration) and reduce the magnitude of high temporal frequency under-sampling artefacts. Trajectories with a sampling point-spread function that changes every frame (e.g. golden angle radial trajectories) can result in high temporal frequency under-sampling artefacts, and so are well suited to this approach. The reconstruction is governed by Eq. (5). ∇ is a finite-difference operator acting on the temporal dimension of each temporal process, and *λ*_∇_ is the corresponding weighting parameter:

(5) $$X,T=argmin_{X,T}(∥\text{E}(\text{X}*\text{T})-\text{d}{∥}_{2}^{2}+\lambda_{\nabla }∥\nabla \text{T}{∥}_{2}^{2})$$

### 2.2 Experimental details

We evaluated the different reconstructions (Tikhonov-constrained, LRP-constrained, smoothness-constrained, k-t FASTER, and k-t PSF) with both retrospectively under-sampled data in various SNR regimes, and with prospectively under-sampled data. The reconstructions are evaluated based on how accurately the spatial, temporal, and functional information is captured across a range of acceleration factors.

#### 2.2.1 Data acquisition

All datasets were generated from a 30s/30s on/off finger-tapping task, and recreated 100 × 100 images with a 2 mm isotropic voxel resolution. An SVD compressed the 32-coil channel to the 8 most dominant components for speed/memory purposes (Zhang et al., 2013; Buehrer et al., 2007). All data were acquired on a 3T system (Prisma, Siemens Healthineers, Erlangen Germany) with informed consent in accordance with local ethics.

**Fig. 2:**
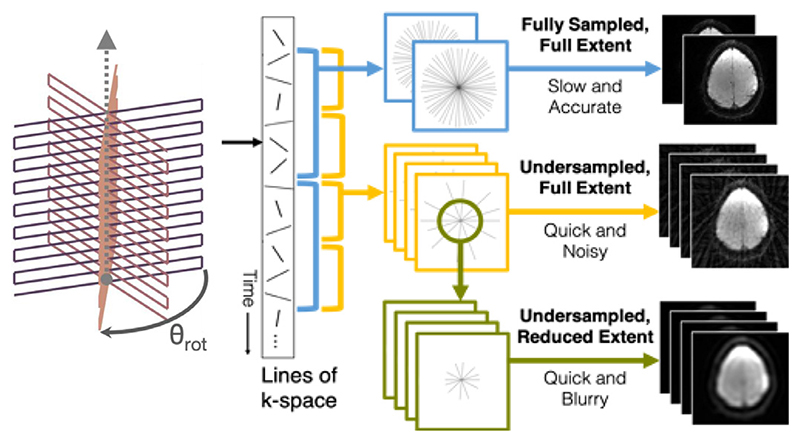
A demonstration of the flexibility of a golden angle sampling scheme, and of the k-space windowing required to create LRP constraints. EPI planes (left) are rotated by ≈ 111.25° around the phase-encoding axis. These rotated planes can then be flexibly combined. If many planes are used (top, blue) then a clean image is easily generated, but at the cost of temporal resolution. If fewer planes are used (middle, yellow) then more images are generated per second, but with an increased number of artefacts. The central part of under-sampled k-space satisfies the Nyquist criterion, even if the full extent of the under-sampled k-space does not. By windowing this central k-space (green, bottom), an accurate low-resolution depiction of the underlying data can be created.

In order to fulfil the non-uniform sampling requirements of the LRP constraints and the changing sampling PSF requirement of the smoothness constraints, all acquisitions in k-space followed the TURBINE trajectory (Graedel et al., 2017; McNab et al., 2009), a 3D hybrid radial-Cartesian EPI sequence which rotates an EPI blade around the phase encoding axis at constant azimuthal increments of the Golden Ratio angle (*π*/Φ ≈ 111.25°) (Winkelmann et al., 2007). This scheme provides a near-uniform radial sampling of k-space from any arbitrary post-hoc combination of consecutive blades, allowing for flexible degrees of acceleration (Fig. 2) (Kim et al., 2011). The under-sampling (or acceleration) factor R is defined here as the ratio of sampling lines required to fully sample k-space to the number of sampling lines acquired. In radial sampling, *R* = 1 requires *π*/2 times more lines than Cartesian sampling.

##### Retrospectively under-sampled datasets

“Retrospective Dataset A” was created by retrospectively resampling each frame of a single fully sampled dataset (300 frames, TR_frame_= 1 s) in k-space with a TURBINE pattern. The original dataset is used as a comparative ground truth, and was acquired as a full volume through a TURBINE acquisition with 20 blades/frame (TR_blade_= 50 ms, TE_blade_ = 30 ms, *R* = 7.85), an acceleration factor shown to produce high fidelity reconstructions with a k-t FASTER approach (Chiew et al., 2016). A single axial slice of one subject with clear bilateral activation was chosen for reconstruction (out of 72 slices in the original dataset). No rank reduction was applied to the original data. The dataset was sampled from a magnitude-only ground truth, with no added noise or phase variation. The retrospective acceleration factors used are *R* = 15.71, 31.42, 39.27, and 52.36 (corresponding to 10, 5, 4, and 3 blades/frame respectively).

“Retrospective Dataset B” was created by adding complex Gaussian noise in k-t space to retrospective dataset A at *R* = 31.42, to highlight the performance difference between the different approaches with additional thermal noise. Noise was added to form new noisy datasets with high (SNR = 100), medium (50), and low (20) SNRs, with the original dataset considered noiseless for the purposes of comparison. For each SNR, five unique instantiations of the noise were added to the underlying data before reconstruction. These values are representative of actual fMRI SNR values (Welvaert and Rosseel (2013)). This additional Gaussian noise only models additive thermal noise as a step towards more realistic data (coherent noise sources such as physiological noise with temporal autocorrelation are not modelled here).

##### Prospectively under-sampled datasets

The following prospectively under-sampled datasets were generated from TURBINE acquisitions. Slices were first reconstructed by performing an inverse FFT along the phase-encode (z) direction before a k-t reconstruction was carried out on each (x-y) k-space plane. The experiment type varies between datasets, but the acquisition parameters were the same in all cases (TR_blade_= 50 ms, TE_blade_= 30 ms, flip angle = 15°, BW = 1786 Hz/px).

“Prospective Dataset A” used a TURBINE acquisition across eight different slices centred on the motor cortex of a single subject. A short experiment (320s, five 30s on/off task epochs) and a long experiment (640s, ten epochs) were carried out consecutively on the same subject. An *R* = 1.05 reconstruction of the long dataset contains enough temporal Degrees-of-Freedom to characterize the underlying functional signal and provide high-quality activation maps, serving as a fully-sampled approximate “ground truth” reference against which the reconstruction of the accelerated short dataset can be separately verified. While ground truths are difficult to establish in prospectively under-sampled datasets, a comparison to either similarly acquired data over a longer timeframe or a moderately under-sampled version of the same dataset can be reinterpreted as a close approximation to the truth for comparative purposes. The different acceleration factors in the short dataset (*R* = 7.85, *R* = 15.71, *R* = 26.18) lead to different temporal resolutions and temporal degrees of freedom, as well as affecting other statistical properties (such as physiological noise variance). While the most general method would reconstruct all eight slices simultaneously to capture shared temporal processes, the extra computational power required for this was not considered worth the benefits, and hence slices were reconstructed independently. The reconstruction details are listed in Table 1.

“Prospective Dataset B” comprises four single-slice datasets taken from separate experiments (all 300 s, five on/off epochs) across two subjects. Two of the acquisitions are centred on the motor cortex, two are centred on the visual cortex. The reconstructions will be labelled Motor 1, Visual 1, Motor 2, and Visual 2 to reflect the location and subject. In this dataset, an *R* = 7.85 k-t FASTER reconstruction (TR_frame_= 1.0 s) is used as a ground truth (an approximation which is justified by the results found from the Prospective Dataset A in Fig. 7), as well as from previous results in the literature (Chiew et al., 2016). This “truth” is only an approximation, and should serve as guidance to a good reconstruction, rather than a definitive measure. Only the highest acceleration factor (*R* = 26.18, TR_frame_= 0.3 s) and best-performing constraints (Tikhonov and Smoothness) from Prospective Dataset A are tested in this dataset, along with k-t FASTER for comparative purposes. The reconstruction details are listed in Table 2.

#### 2.2.2 Selection of reconstruction parameters

**Fig. 3:**
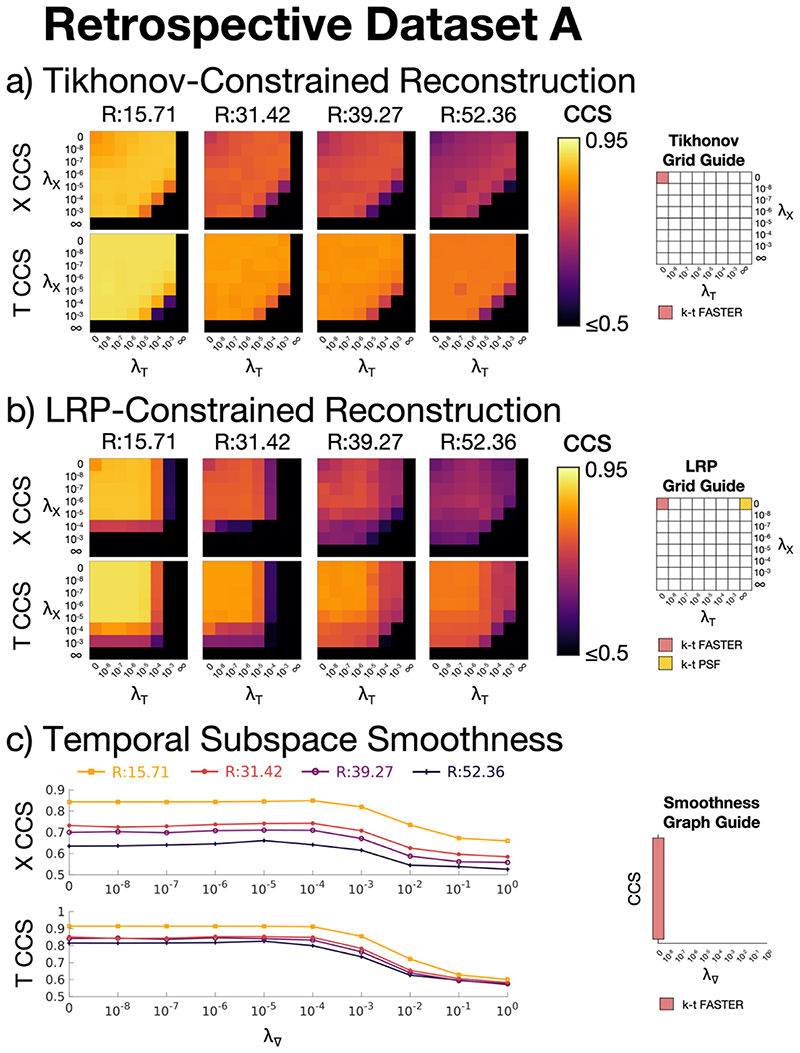
The canonical correlation scores (CCS) of retrospective dataset A vs a ground truth for a): Tikhonov-constrained reconstructions, b): LRP-constrained reconstructions, c): Temporal Subspace Smoothness reconstructions. X CCS and T CCS refer to the spatial and temporal Canonical Correlation Scores respectively. The acceleration factors shown are: *R* = 15.71 (10 blades/frame), *R* = 31.42 (5 blades/frame), *R* = 39.27 (4 blades/frame), and *R* = 52.36 (3 blades/frame). The *λ* values encoding the pre-existing k-t FASTER and k-t PSF methods are shown on the right for each constraint.

A logarithmic grid search over potential *λ*_X_ and *λ*_T_ candidates was carried out for all datasets, constraints, and acceleration factors. The grid search for retrospective dataset A is shown in Fig. 3 to demonstrate the typical effects of varying *λ* on the reconstructed spatial and temporal information for the different constraints, with boundary cases shown for *λ* = 0 (zero prior influence) and *λ* = ∞ (the solution is fixed to the prior). The special boundary case of (*λ_X_* = 0, *λ_T_* = 0) defines k-t FASTER for all constraints and the special case of (*λ_x_* = 0, *λ_T_* = ∞) defines k-t PSF with LRP constraints. As the smoothness constraints rely on a single weighting parameter (*λ*_∇_, the results are shown as a line graph.

The reconstruction rank was fixed at 16 for all reconstructions, including priors (this rank matches a value used in recent literature for low-rank task fMRI Chiew et al., 2018). A variety of acceleration factors were tested. The convergence criterion was defined as the normalized gradient for the whole cost function *CF* (Eq. (6)), evaluated after the temporal subproblem optimization for iteration number *i*.

(6) $$\frac{\left|CF_{i}-CF_{i-1}\right|}{CF_{i}}<\varepsilon$$

A criterion of *ε* = 10^−5^ was used for both retrospective datasets, which was chosen as the value at which a k-t FASTER reconstruction with different random initializations was found to converge to identical subspaces. For the prospective datasets, *ε* = 10^−3^ was found to be more optimal. This lower convergence criterion was found to produce slightly improved statistical parameter maps (defined using the metrics of Section 2.2.3), which may be a result of overfitting occurring at the more precise criterion used in both retrospective datasets. The different criteria chosen here were selected to ensure a very high level of agreement regardless of the initialization, and were chosen using the k-t FASTER reconstruction without additional subspace constraints. Future experiments may well benefit from more liberal criteria to enable faster reconstruction, without necessarily experiencing any loss in reconstruction quality.

#### 2.2.3 Evaluation and fMRI analysis

Reconstruction image quality can be difficult to determine (Wang et al., 2004), with more incoherent (‘noise-like’) artefacts usually preferable to coherent artefacts, and the first component of the subspace dominating most image quality metrics (such as root mean square error or structural similarity index). Spatial artefacts can also make conventional metrics like SNR (or simple measures of noise) harder to quantify.

Instead, the spatial and temporal subspaces were directly compared to the retrospective ground truth subspaces using canonical correlation analysis. Canonical correlation measures the cosine of the principal angles (the alignment) between subspaces (Knyazev and Argentati (2002)), with higher values reflecting more aligned subspaces, and a value equal to the rank of the subspace (16 in all cases) demonstrating complete alignment. A Canonical Correlation Score (CCS) was created by dividing the canonical correlation by the maximal rank of the decomposed matrices, providing a normalized metric measuring the alignment of the subspaces. X CCS and T CCS respectively refer to the CCS for spatial and temporal subspace analyses. As a subspace alignment metric, CCS does not account for the magnitude of the estimated components, only their relative alignment. This potential shortcoming is accepted for two reasons: firstly, the data consistency term will generally ensure that the relative magnitude of the signal is well captured and secondly, any ICA analysis run on the data will also be scale-independent (Hyvärinen and Oja (2000)). CCS cannot be used where dimensionality varies between two, so this metric was only used in evaluations of the retrospective sampling comparisons.

For all datasets, task fMRI analysis was performed in FEAT (FSL) (Smith et al., 2004). Because the fMRI is smooth, increasing temporal resolution in data also increases the autocorrelation of the measured signal. Where this results in smooth residuals (e.g. due to physiological noise or imperfect modelling), assuming a known null distribution can inflate calculated z-statistics (Feinberg et al., 2010). The resulting z-statistic maps were null-corrected using mixture modelling (Beckmann and Smith (2004)) to account for residual autocorrelation, and deviations in effective temporal degrees of freedom that arise from high acceleration factors in the prospective datasets. Reconstructed prospective data are aligned to the ground truth reference using FLIRT (Jenkinson and Smith (2001)) prior to analysis. Receiver Operating Characteristic (ROC) curves were calculated to measure the false positive rate (FPR) against true positive rate when comparing the reconstructions against the activation map of a fully sampled reconstruction. A threshold of *z*≥3.1 was used to threshold the retrospective truth, *z*≥4.8 was used for Prospective Dataset A, *z*≥4.0 was used for Motor 1/Visual 1 in Prospective Dataset B, and *z*≥2.7 was used for Motor 2/Visual 2 in Prospective Dataset B (these values were selected heuristically based on anatomical veracity of known regions of expected activation). *Z*-statistic parameter maps are shown at a false positive rate of 0.0015 in order to facilitate visualization. The ROC curves will be focussed on low FPRs, as the z-statistic corresponding to high FPRs would never be used in studies. The Area Under the Curve (AUC) of the full ROC curve allows for a simple comparison of many reconstructions, but the underlying z-statistic maps also provide valuable information as to the spatial location of false positives and false negatives.

## 3 Results

Optimal values of *λ_X_*, *λ_T_,* and *λ_∇_* are evaluated for each dataset, method, and acceleration factor, and then the optimized reconstructions are evaluated against the reconstructions using the k-t FASTER and k-t PSF methods. The optima are selected using a heuristic combination of the CCSs (retrospective datasets only), ROC AUCs, and qualitative assessments of z-statistic activation maps.

### 3.1 Retrospective Dataset A results

The influence of *λ_X_* and *λ_T_* on the recovered temporal and spatial components for different constraints is shown in Fig. 3. The LRP constraints are defined by a peak in spatial CCS and a broad plateau in temporal CCS (although the gradient is quite shallow near the peak). The Tikhonov constraints were defined by a line of peak values normal to *λ_X_* = *λ_T_*, suggesting a 1D search could suffice to find an optimal *λ* pairing. For Tikhonov and LRP constraints, the upper-left-hand corner of every *λ* grid represents k-t FASTER, and the far left point represents k-t FASTER in the 1D plot. The upper-right-hand corner of the LRP constraint *λ* grids represents k-t PSF. The optimal *λ* values are shown in Table 3, and were constant across acceleration factors, except for the highest acceleration factor (*R* = 52.36).

**Fig. 4:**
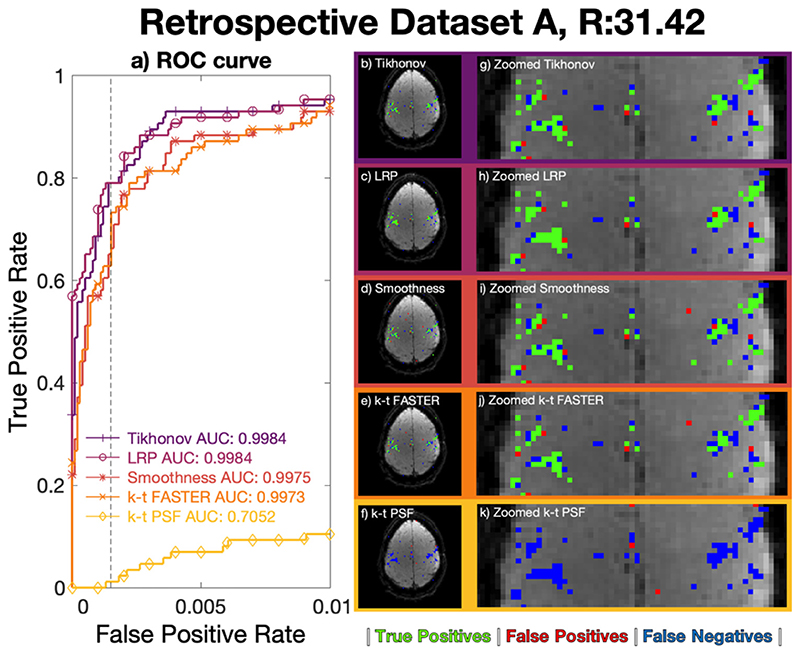
*R* = 31.42 (5 blades/frame) retrospective dataset A reconstructions. a) ROC curves, legend lists full curve AUC. b)-f) Activation maps using a z-statistic corresponding to an FPR of 0.15%. g)-k) A medial zoom of the associated activation maps. b/g) Tikhonov: *λ_X_* = 10^−5^, *λ_T_* = 10^−5^, c/h) LRP: *λ_X_* = 10^−5^, *λ_T_* = 10^−5^, d/i) Temporal subspace smoothness: *λ*_∇_ = 10^−5^, e/j) k-t FASTER, f/k) k-t PSF. Maps b)-k) use green true positive pixels, red false positives, and blue false negatives.

**Fig. 5:**
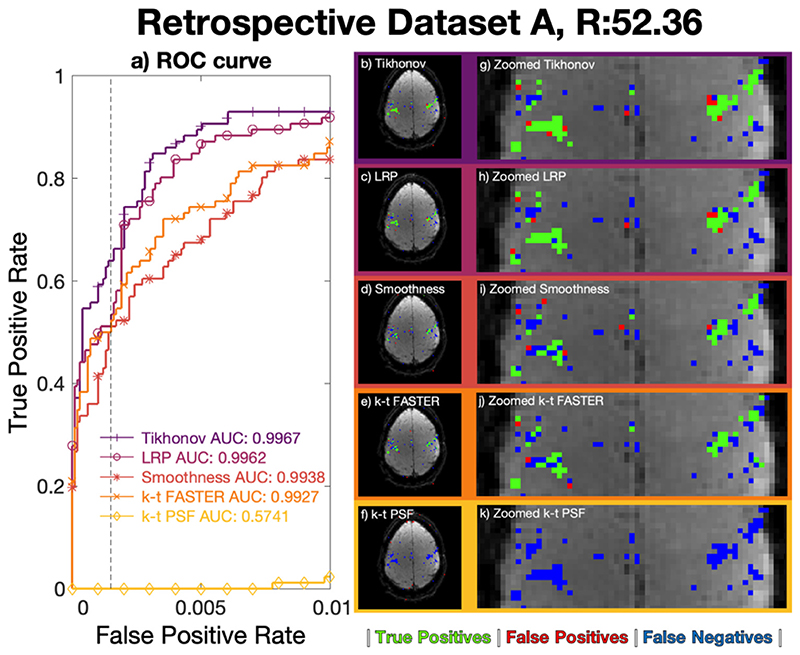
*R* = 52.36 (3 blades/frame) retrospective dataset A reconstructions. a) ROC curves, legend lists full curve AUC. b)-f) Activation maps using a z-statistic corresponding to an FPR of 0.15%. g)-k) A medial zoom of the associated activation maps. b/g) Tikhonov: *λ_X_* = 10^−5^, *λ_T_* = 10^−5^, c/h) LRP: *λ_X_* = 10^−4^, *λ_T_* = 10^−6^, d/i) Temporal subspace smoothness: *λ*_∇_= 10^−4^, e/j) k-t FASTER, f/k) k-t PSF. Maps b)-k) use green true positive pixels, red false positives, and blue false negatives.

*Z*-statistic activation maps were derived for all approaches using the optimized *λ* values at *R* = 31.42 (Fig. 4) and *R* = 52.36 (Fig. 5), and are overlaid on the mean dataset image. The ROC curves and activation maps are consistent with the results of Fig. 3, with the Tikhonov and LRP constraints performing better than the other k-t methods at both acceleration factors, albeit with the Tikhonov regularization marginally outperforming LRP-constrained reconstruction at *R* = 52.36. The cleanness of the dataset appeared to allow very high reconstruction factors which were not found to be possible in more realistic data.

### 3.2 Retrospective Dataset B results

Optimal *λ* was found to increase as SNR decreased for Tikhonov and LRP results. The following values were used for both Tikhonov and LRP constraints: high SNR (SNR = 100, *λ_X_* = 10^−4^, *λ_T_* = 10^−5^); medium SNR (SNR = 50, *λ_X_* = 10^−4^, *λ_T_* = 10^−4^); low SNR (SNR = 20, *λ_X_* = 10^−3^, *λ_T_* = 10^−4^). The temporal subspace smoothness results used *λ*_∇_= 10^−4^ in all cases, although the variation in results was small for 10^−4^ ≤ *λ*_∇_ ≤ 10^−1^.

**Fig. 6:**
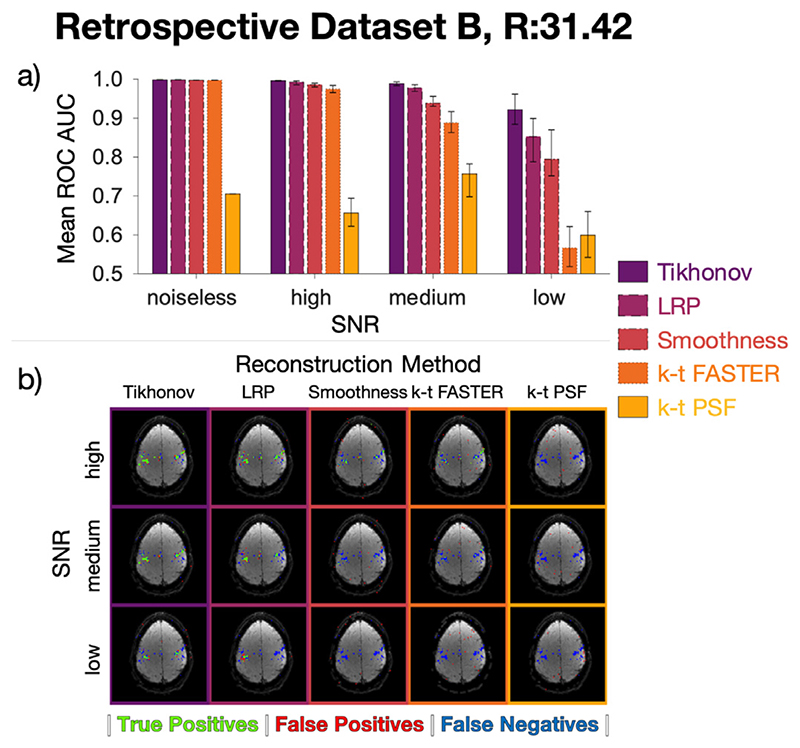
**6a:** Retrospective dataset B reconstruction AUC results. Each bar represents the mean AUC of five different instantiations of Gaussian noise in k-t space at a specific SNR for a specific reconstruction method, except for the lefthand set, which represent a single noiseless reconstruction. The error bars show the range of AUC values. **6b:** An example activation map at each noise value for each reconstruction method. See Supplementary Figs. 3–5 for the full set of activation maps and the individual ROC curves. As with Figs. 4-5, green pixels represent true positives, red pixels represent false positives, blue pixels represent false negatives. The z-statistics threshold yielded a false positive rate of 0.15%.

The mean AUC of the noisy parameter map ROCs compared to a noiseless truth are summarized in Fig. 6a, with all reconstructions losing fidelity as SNR decreased. The noiseless reconstructions are equivalent to the data shown in Fig. 4. Maps comparing thresholded z-stat maps with the ground truth for each method are shown in Fig. 6b, with full visualizations of all reconstruction activation maps and ROC curves shown in Supplementary Figs. 3–5. The maps are overlaid on top of the mean functional image of each reconstruction. In t-tests performed between the different constraints within the three non-noiseless SNRs, all reconstructions within an acceleration factor were significantly different (*p* < 0.05) except Tikhonov vs LRP at high SNR, k-t FASTER vs k-t PSF at low SNR, and LRP vs Smoothness at low SNR

Tikhonov-constrained reconstruction outperformed all other methods, identifying plausible activity even at the lowest SNR tested. LRP and temporal smoothness constraints represent improvements on the previously proposed techniques (k-t FASTER and PSF), with all constrained results better than all k-t FASTER results at medium and low SNR. The k-t FASTER approach appears highly susceptible to noise, with a roughly equivalent noiseless AUC score to the other methods at *R* = 31.42 (Fig. 5) rapidly decreasing as SNR decreased. The k-t PSF approach failed to capture activation even for the noiseless simulated dataset at this acceleration factor.

### 3.3 Prospective Dataset A results

This section presents results on the first prospectively under-sampled (“real”) experiments, with three different acceleration factors tested: *R* = 7.85 (20 blades/frame), *R* = 15.71 (10 blades/frame), and *R* = 26.18 (6 blades/frame). The optimal *λ* values were found to be dependant on both R and the chosen constraint in the prospective dataset (the distribution of reconstruction scores with respect to *λ* were similar to Fig. 3, and so are not shown here). The only exception is that the LRPs were less dependant on *λ_T_*, with a broader range of values producing scores close to the optimum. Optimal *λ* values for this dataset are shown in Table 4.

**Fig. 7:**
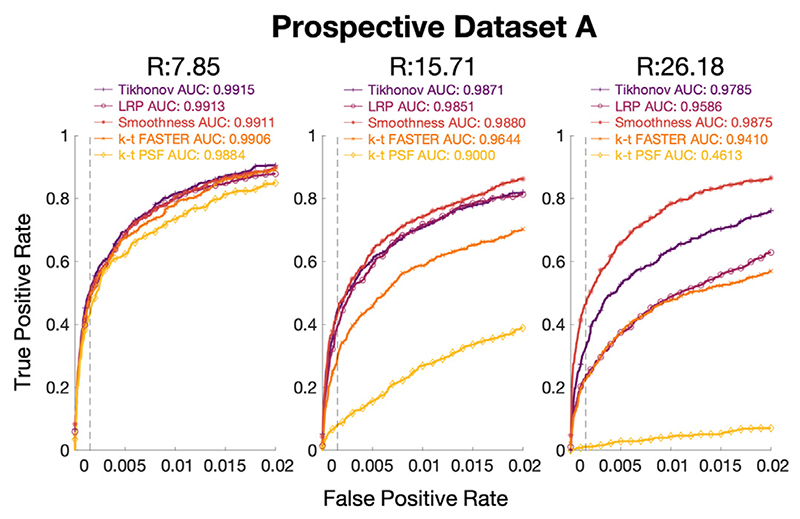
The ROC curves across eight slices for a) *R* = 7.85 (20 blades/frame), b) *R* = 15.71 (10 blades/frame), and c) *R* = 26.18 (6 blades/frame). The ground truth is the long dataset taken under similar experimental conditions, at a threshold of *z* ≥ 4.8. The false-positive rate is shown on the x-axis up to 0.02, in order to allow visualization of the analytically relevant representation of the activation maps.

**Fig. 8:**
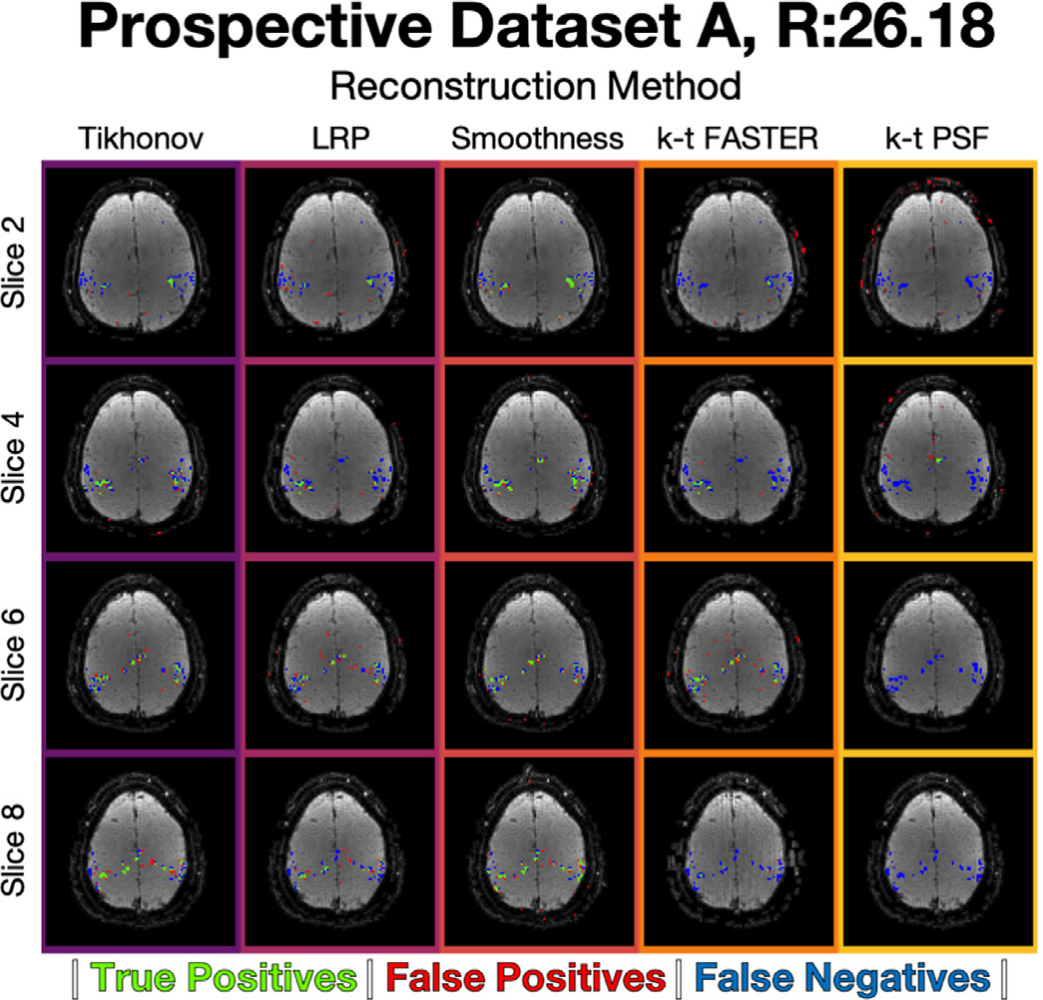
Prospective Dataset A, *R* = 26.18. The activation maps for every second slice of the reconstruction, at a threshold defined by a 0.15% volumetric false positive rate. Supplementary Fig. 8 shows the activation maps of all slices.

The ROC curves for the optimal *λ* at each acceleration factor for each method are shown in Fig. 7. The activation maps for every second slice of the *R* = 26.18 results are shown in Fig. 8. The full selection of activation maps for all slices and acceleration factors can be seen in Supplementary Figs. 6–8. All activation maps are overlaid on the mean functional image, with an example function image for each *R* = 26.18 dataset shown in Supplementary Fig. 9. The effects of the different reconstruction approaches on tSNR and signal autocorrelation are shown in Supplementary Figs. 10 and 11 respectively.

**Fig. 9:**
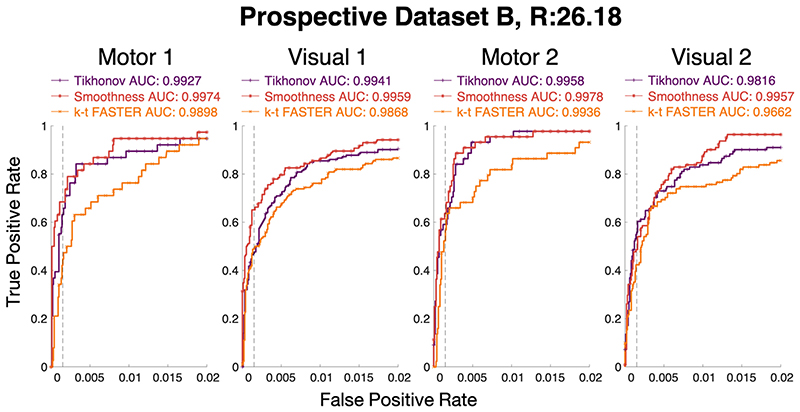
The ROC curves for each different slice of Prospective Dataset B at *R* = 26.18 (6 blades/frame), compared to an *R* = 7.85 k-t FASTER reconstruction of the same slice thresholded at either *z* ≥ 4.0 (Motor 1/Visual 1) or *z* ≥ 2.7 (Motor 2/Visual 2). The false-positive rate is shown on the x-axis up to 0.02, in order to allow visualization of the analytically relevant representation of the activation maps.

At the lower acceleration factor (*R* = 7.85), all approaches appear approximately equivalent, with k-t PSF performing worst with AUC = 0.9884 and all other methods having AUC > 0.99. At the medium acceleration factors (*R* = 15.71), the soft subspace constraints outperformed k-t FASTER (AUC = 0.9644) and k-t PSF (AUC = 0.9000) with AUC > 0.98. At the high acceleration factor (*R* = 26.18, Fig. 9), the Tikhonov-constrained results and smoothness results outperformed all other methods with AUCs of 0.9785 and 0.9875 respectively, and the LRP constrained method (AUC = 0.9586) performing similar to k-t FASTER (AUC = 0.9410) at this acceleration factor. Here, the smoothness constraints outperformed the Tikhonov constraints by a score of 0.09, whereas the Tikhonov constraints either performed equivalently or outperformed the smoothness constraints in all previous scenarios.

### 3.4 Prospective Dataset B results

This section presents results on the second set of prospectively under-sampled experiments, with only one acceleration factor tested: *R* = 26.18 (6 blades/frame). The ground truth is taken as the *R* = 7.85 (20 blades/frame) k-t FASTER reconstruction of a slice for comparative purposes. The optimal *λ* values for the smoothness constraint were found to be consistent with Prospective Dataset A at this acceleration factor, whereas the optimal Tikhonov *λ* values varied between slices (Table 5).

**Fig. 10:**
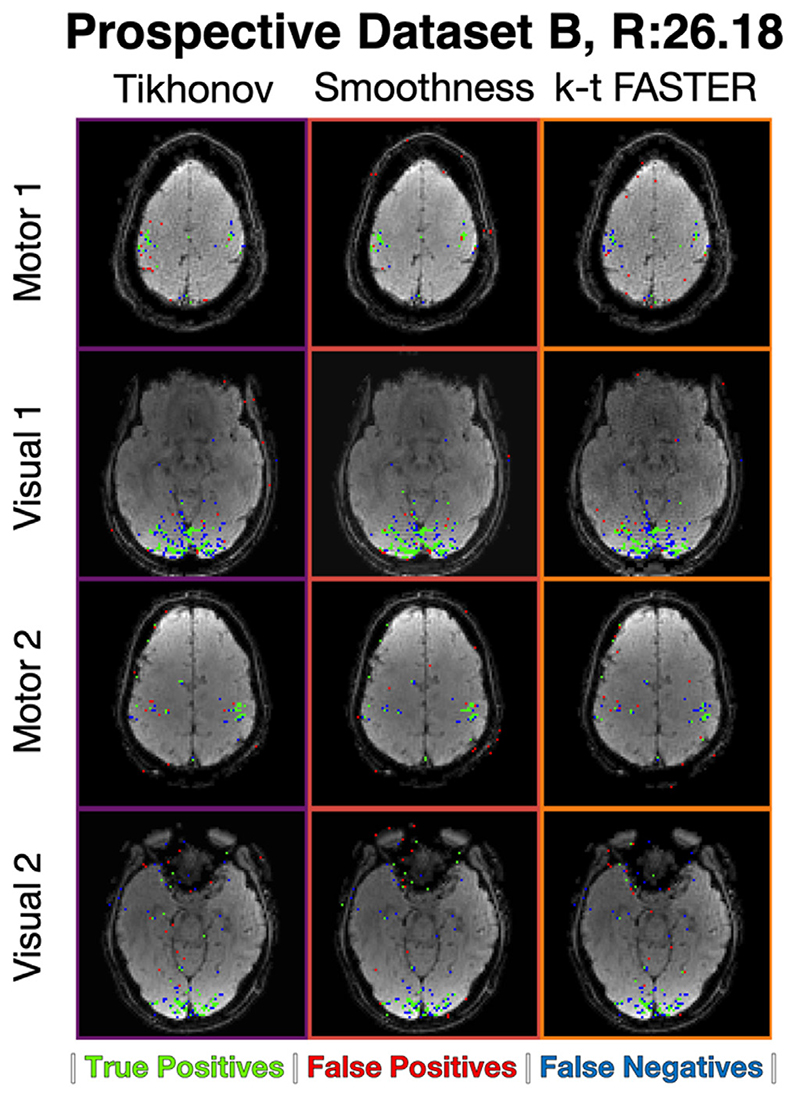
Prospective Dataset B, *R* = 26.18. The activation maps for each reconstruction, at a threshold defined by a 0.15% volumetric false positive rate. The background brain is the mean temporal image for that reconstruction.

The ROC curves for the optimal *λ* at each acceleration factor for each method are shown in Fig. 9. The smoothness constraint appeared to produce the best ROC AUC score in all cases, with Tikhonov outperforming the k-t FASTER approach. The activation maps shown in Fig. 10. In some cases, the specific 0.15% false positive rate threshold meant that a method with a lower ROC AUC score produced a more accurate map. The underlying mean image is generally cleaner in the two constrained methods when compared to k-t FASTER.

## 4 Discussion

This study demonstrates the impact of three different L2-based constraints in a global low-rank optimization framework for accelerated fMRI data reconstruction. In instances of high acceleration or low SNR, the constrained approaches are able to better identify true regions of activation in a finger-tapping study, as well as producing solutions that more closely map to the spatial and temporal subspaces of a ground truth. These results highlight the viability of non-linear reconstruction frameworks in fMRI that do not rely explicitly on sparse modelling of the BOLD signals.

### 4.1 Comparison between methods

Across the different evaluated datasets a clear trend emerged: the addition of soft subspace-constraints to the k-t FASTER formulation produces improved subspace alignment and ROC AUC scores at high acceleration/low SNR. Collectively, the qualitative and quantitative metrics reveal that very high acceleration factors are possible with these soft constrained-subspace low-rank approaches, in the right conditions. The conditions tested in this paper show that the fMRI signal of interest can be represented by a small number of high-variance components, as elicited with a finger-tapping motor task experiment. The effectiveness of this approach in other, lower-variance examples such as resting-state fMRI or more subtle task fMRI experiments remains to be seen.

The non-linear reconstruction framework only aimed to recover the first 16 components in a low-rank representation of the signal, resulting in feasible reconstructions at very high acceleration due to the reduced matrix degrees of freedom in the estimated output. The high acceleration factors in the retrospective dataset A (e.g. *R* = 52.36) were chosen to differentiate between different constraints, and are not considered representative of realistic acceleration factors. The acceleration factors reported for the prospective datasets (*R* = 26.18) are considerably higher than those reported in previous studies of low-rank fMRI reconstruction using realistic data, which is facilitated largely by the additional soft subspace-constraints. However, as evidenced in Supplementary Fig. 11, some care must be taken in interpreting the actual temporal resolution of the reconstructions, particularly with the temporal smoothness constraints, and effective acceleration factors may be lower than the nominal reported under-sampling factors. To account for this, effective temporal degrees of freedom resulting from temporal smoothing constrained reconstruction can be estimated analytically (Chiew and Miller, Dec. 2019), or corrected for using a statistical mixture modelling procedure to normalize the null-distribution of the *z*-statistics (Beckmann and Smith (2004)).

The Tikhonov constraints produced high fidelity reconstructions in both retrospective and prospective under-sampling, even at acceleration factors or SNR levels where other methods began to fail (e.g. the prospective *R* = 26.18/TR = 0.3 s results, or the low SNR retrospective dataset B results). Additionally, Tikhonov-constrained reconstructions were the fastest to reconstruct out of all the softly constrained reconstructions while its optimal *λ* pairing could be found through a 1-D parameter search only - reducing the dimensionality of the design constraints.

However, the Tikhonov reconstructions were outperformed by the temporal subspace smoothness approach in the reconstructions of the prospectively under-sampled data, despite that same smoothness approach only providing a relatively small improvement over k-t FASTER in both retrospective datasets. However, the retrospective datasets were constructed under conditions that were favourable for k-t FASTER, without any additional phase modulations or physiological noise (beyond what was in the original dataset). The scale of improvement is also worth noting, with the AUC scores showing Tikhonov outperforming smoothness by an absolute value of +0.3% in the most discriminatory result of retrospective dataset A (*R* = 52.36, 0.9967 vs 0.9938), but smoothness outperforming Tikhonov by +0.9% in the highest acceleration factor tested in the Prospective Data A (*R* = 26.18, 0.9875 vs 0.9785), and an average +0.6% improvement at the same acceleration factor for Prospective Dataset B. This smoothness improvement is in addition to the improvement the Tikhonov approach manages over all other methods (+3.75%/+0.7% total over k-t FASTER in Prospective Dataset A/B), while also occurring in the dataset most representative of real data. Both Tikhonov and Smoothness constraints also generally improve the mean functional image compared to k-t FASTER (the background of Fig. 8/Fig. 10 over which the activation maps are overlain). The outstanding question from these findings is then whether all real-data reconstructions favour smoothing constraints, or are there a set of conditions in real data that would favour Tikhonov constraints?

The low-resolution priors were unable to match the performance of the Tikhonov constraints in any dataset, nor the temporal smoothness in Prospective Dataset A. The false positives in the LRP-constrained z-stat maps were localized close to the area of interest, indicating the influence of the prior on the resulting potential reduction in effective spatial resolution. By comparison, at lower SNR the k-t FASTER approach produced false positives which were less localized to voxels adjacent to true positive activations. As a generalization of the k-t PSF approach, this may reflect the intrinsic limitation of generating priors from low-resolution training data for constraining a high-resolution reconstruction. Furthermore, reconstruction times for the LRP constrained reconstructions were the longest by far.

The k-t PSF method did well at *R* = 7.85 in the real prospective data, and has not to our knowledge been previously tested without sparsity constraints in an fMRI framework. However, the formulation of k-t PSF used in this paper did not produce robust solutions in the other datasets or at the higher acceleration factors tested This is also consistent with the performance of the low-resolution prior method, where both methods that constrained the reconstruction based on a low-spatial resolution temporal basis were not as successful as the other constraints in under-sampled signal recovery.

The optimal regularization factors varied due to a number of factors. Tikhonov/LRP *λ* values were strongly dependant on SNR in Retrospective Dataset B, weakly dependant on R within a dataset, and varied between datasets at a given R. The smoothness weighting varied strongly with R in Prospective Dataset A, but was found to be consistent for all Prospective Dataset B reconstructions at that R, and was also consistent across SNRs in Retrospective Dataset B. At *R* = 26.18, Tikhonov values of *λ_X_ =* 10^−2^, *λ_T_* = 10^−1^ and Smoothness values of *λ_∇_* = 10^+2^ were the most common optimal regularization parameters in prospective reconstructions under the experimental parameters tested in this paper. It is clear that a soft constraint can help guide the dataset to improved reconstruction scores, but as with many regularization methods, the identification of optimal *λ* parameters will require some care.

### 4.2 Limitations and future work

One limitation of this work is the small sample of datasets used to evaluate the methods, and further testing on additional datasets with physiological noise models or other confounding factors would be needed to establish robustness. This would allow more insight into the robustness of the Tikhonov and smoothness constraints, the optimal *λ* values, and the impact of coherent noise contamination or auto-regressive noise properties on the different approaches. In addition, further dataset testing could assess the impact of motion. Motion can violate the low-rank assumptions in fMRI, with motion-related variance swamping BOLD fluctuations, and so adequate motion-correction is required. However, a major challenge is that this effect cannot be corrected post-hoc using conventional time-series registration, but needs to correct the k-space data prior to low-rank reconstruction. The data collected for this study was performed on healthy volunteers with very little apparent motion, although the TURBINE k-space trajectory enables motion correction using low spatial resolution navigators (Graedel et al., 2017). One solution could involve combining TURBINE’s self-navigation capabilities with a joint estimation of the subspaces and motion parameters, leveraging an assumption that a motion-free reconstruction would have the lowest rank or nuclear norm. While the TURBINE acquisition scheme was used to help fulfil the non-uniform sampling density requirement of the LRP constraints, alternative sampling schemes could also be tested to explore how well the smoothness and Tikhonov constraints generalize. Aside from the potential motion-correction benefits, TURBINE was chosen for this paper due both to its inherent flexibility in acceleration and the noise-like aliasing produced in under-sampling. In contrast, standard approaches with Cartesian sampling, like 3D EPI or blipped-CAIPI SMS EPI, do not provide sufficient sampling incoherence for effective use of low-rank constraints (Chiew et al., 2016). However, investigation of novel sampling trajectories for non-linear reconstruction models is a topic of increasing interest (Lazarus et al., 2019), and it would be interesting to determine more optimal trajectories for these low-rank reconstruction models.

The joint-optimization of two subspaces in alternating minimization provides a flexible reconstruction framework, but could benefit from speeding up. The slowest reconstructions took up to 10 s of hours per slice for both Tikhonov and smoothness-constrained reconstruction (Table 2). While Toeplitz Embedding was used to speed up iterative use of the NUFFT (Ahmad et al., 2011; Chan and Ng (1996)), the reconstruction code has not been optimized for speed and these computation times could likely be reduced significantly. In addition to code optimization, subproblem parameters such as the convergence factor *ε* and the number of internal iterations in each linear subproblem (see Supplementary Fig. 2) were both chosen to be deliberately conservative for this exploratory analysis and could be fine-tuned for faster reconstructions in future.

## 5 Conclusions

Low-rank reconstructions in fMRI can benefit from additional regularization, particularly at high acceleration factors or in low-SNR regimes. The L2-based constrained-subspace approaches studied here were shown to improve upon methods like k-t FASTER in realistic fMRI data at acceleration factors of *R* > 10, although there is an associated increase in reconstruction time as currently implemented. The improvements with the soft subspace constraints were most apparent at the highest acceleration factor tested (*R* = 26, nominal TR = 0.3), and particularly pronounced for the Tikhonov constraints and temporal smoothness constraints.

## Supplementary Material

Supplementary material associated with this article can be found, in the online version, at doi: 10.1016/j.neuroimage.2021.118235.

Supplementary Materials

## Figures and Tables

**Table 1:** The reconstruction details for the different acceleration factors used in reconstructing Prospective Dataset A.

Dataset	Blades	TR_frame_ (s)	R	$\frac{\text{Blades}}{\text{Frames}}$	Frames
**Long**	12,800	7.5	1.05	150	85
**Short**	6400	1.0	7.85	20	320
**Short**	6400	0.5	15.71	10	640
**Short**	6400	0.3	26.18	6	1066

**Table 2:** The reconstruction details for the different acceleration factors used in reconstructing Prospective Dataset B.

Name	Blades	TR_frame_ (s)	R	$\frac{\text{Blades}}{\text{Frames}}$	Frames
Motor 1	6000	0.3	26.18	6	1000
Visual 1	6000	0.3	26.18	6	1000
Motor 2	6000	0.3	26.18	6	1000
Visual 2	6000	0.3	26.18	6	1000

**Table 3:** The optimal 2 values for each method in retrospective dataset A. Results within 0.001 of the best ROC AUC score and 0.01 of the best CCS values are shown in bold.

R	Method	λ_X_	λ_T_	λ_V_	X CCS	T CCS	ROC AUC
**15.71**	Tikhonov	10^−5^	10^−5^	0	**0.89**	**0.91**	**0.9983**
	LRP	10^−5^	10^−5^	0	**0.88**	**0.91**	**0.9985**
	Smoothness	0	0	10^−5^	0.85	**0.91**	**0.9983**
	k-t FASTER	0	0	0	0.84	**0.91**	**0.9983**
	k-t PSF	0	∞	0	0.34	0.28	0.8956
**31.42**	Tikhonov	10^−5^	10^−5^	0	**0.80**	**0.85**	**0.9984**
	LRP	10^−5^	10^−5^	0	0.78	0.82	**0.9984**
	Smoothness	0	0	10^−5^	0.74	**0.85**	**0.9975**
	k-t FASTER	0	0	0	0.73	**0.85**	0.9973
	k-t PSF	0	∞	0	0.22	0.20	0.7052
**39.27**	Tikhonov	10^−5^	10^−5^	0	**0.76**	**0.83**	**0.9974**
	LRP	10^−5^	10^−5^	0	0.72	0.78	**0.9968**
	Smoothness	0	0	10^−5^	0.71	**0.84**	0.9956
	k-t FASTER	0	0	0	0.70	**0.84**	0.9956
	k-t PSF	0	∞	0	0.21	0.22	0.5213
**52.36**	Tikhonov	10^−5^	10^−5^	0	**0.73**	**0.82**	**0.9967**
	LRP	10^−4^	10^−6^	0	0.67	0.78	**0.9962**
	Smoothness	0	0	10^−4^	0.65	0.80	0.9938
	k-t FASTER	0	0	0	0.63	**0.81**	0.9927
	k-t PSF	0	∞	0	0.21	0.23	0.5741

**Table 4:** The optimum λ values in Prospective Dataset A for each constraint at each acceleration factor. The time in brackets shows the split between the time taken to generate the priors and the final reconstruction. Results with the shortest reconstruction time or within 0.001 of the best ROC AUC score are shown in bold.

R	$\frac{\text{Blades}}{\text{Frames}}$	Method	λ_T_	λ_v_	λ_∇_	Mean Recon Time (hours)	ROC AUC
**7.85**	20	Tikhonov	10^−1^	10^−2^	0	2.9	**0.9915**
		LRP	10^−1^	10^−7^	0	(1.7 + 1.6) 3.3	**0.9913**
		Smoothness	0	0	10^−3^	**1.4**	**0.9911**
		k-t FASTER	0	0	0	**1.4**	**0.9906**
		k-t PSF	0	∞	0	(1.7 + 0.3) 2.0	0.9884
**15.71**	10	Tikhonov	10^−1^	10^−2^	0	6.3	**0.9871**
		LRP	10^−1^	10^−3^	0	(26.9 + 6.4) 33.3	0.9851
		Smoothness	0	0	_10_+1	11.2	**0.9880**
		k-t FASTER	0	0	0	**5.8**	0.9644
		k-t PSF	0	∞	0	(26.9 + 0.3) 27.2	0.9000
**26.18**	6	Tikhonov	10^−2^	10^−1^	0	**11.6**	0.9785
		LRP	10^−3^	10^−7^	0	(192.3 + 11.3) 203.6	0.9586
		Smoothness	0	0	10+2	29.6	**0.9875**
		k-t FASTER	0	0	0	13.0	0.9410
		k-t PSF	0	∞	0	(192.3 + 0.3) 192.6	0.4613

**Table 5:** The optimum 2 values in Prospective Dataset B for each constraint at each acceleration factor. The reconstructions here were done on varying numbers of cores due to computation constraints, and so timings are not shown. Results with the best ROC AUC score are shown in bold.

Name	R	$\frac{\text{Blades}}{\text{Frames}}$	Method	λ_X_	λ_T_	λ_V_	ROC AUC
Motor 1	26.18	6	Tikhonov	10^−5^	10^−4^	0	0.9927
			Smoothness	0	0	10^+2^	**0.9974**
			k-t FASTER	0	0	0	0.9898
Visual 1	26.18	6	Tikhonov	10^−3^	10^−2^	0	0.9941
			Smoothness	0	0	10^+2^	**0.9959**
			k-t FASTER	0	0	0	0.9868
Motor 2	26.18	6	Tikhonov	10^−1^	10^−1^	0	0.9958
			Smoothness	0	0	10^+2^	**0.9978**
			k-t FASTER	0	0	0	0.9936
Visual 2	26.18	6	Tikhonov	10^−2^	10^−1^	0	0.9816
			Smoothness	0	0	10^+2^	**0.9957**
			k-t FASTER	0	0	0	0.9662
