# Supplementary material for: Subspace-constrained approaches to low-rank fMRI acceleration
Source: Neuroimage. Author manuscript; Available in PMC 2021 Oct 13. (PMC7611820; doi:10.1016/j.neuroimage.2021.118235)
Supplement: Supplementary Materials [file EMS136203-supplement-Supplementary_Materials.docx]

**Appendix A: Pseudocode**

*Input*d: multicoil under-sampled k-t fMRI data
E: Sampling and multi-coil encoding operator

λ_X_: spatial regularization weighting factor
λ_T_: temporal regularization weighting factor

λ_∇_: temporal smoothness regularization weighting factor

*%Initialize*
$X_{{prior0}_{col\_1}}$: Temporal Mean (The average image over all time)
$X_{\mathrm{prior}0_{cols\_2:r}}$: 0
$T_{\mathrm{prior}0}$ $T_{prior0}$: Randomly orthogonal rows

*%Create Priors*

$$d_{\mathrm{win}}=\mathrm{window}(d)$$

**while** not converged **do**
$X_{\mathrm{prior}_{i+1}}$🡨${argmin}_{X_{\mathrm{prior}}}\left( \left\| E\left( X_{\mathrm{prior}} T_{\mathrm{prior}_{i}}’ \right)- d_{\mathrm{win}} \right\|_{2}^{2} \right)$
$T_{\mathrm{prior}_{i+1}}$🡨${argmin}_{T_{\mathrm{prior}}}\left( \left\| E( X_{\mathrm{prior}_{i+1}}T_{\mathrm{prior}}’ ) - d_{\mathrm{win}} \right\|_{2}^{2} \right)$
end **while**

*%Final Reconstruction*

$X_{0}$ = $X_{\mathrm{prior}}$
$T_{0}$ = $T_{\mathrm{prior}}$

**while** not converged **do**
$X_{i+1}$🡨${argmin}_{X}\left( \left\| E(X T_{i}’)-d \right\|_{2}^{2}+\lambda_{X}\left\| X-X_{\mathrm{prior}} \right\|_{2}^{2} \right)$
$T_{i+1}$🡨${argmin}_{T}\left( \left\| E( X_{i+1}T’ )-d \right\|_{2}^{2}{+ \lambda_{T}\left\| T-T_{prior} \right\|}_{2}^{2} {+ \lambda}_{\nabla}\left\| \nabla T \right\|_{2}^{2} \right)$
end **while**

*%Output*D = X*T’: Final reconstructed x-t fMRI data

Test code for running the main algorithm of this paper can be found at <https://github.com/harrytmason/constrained-lowrank-recon>, and the data can be downloaded from the Oxford Research Archives, <https://ora.ox.ac.uk/objects/uuid:78743195-4217-4e83-a749-d74941b3b2ac>.

**Appendix B: Implementation Details**

There are a few ways to tackle a k-t space reconstruction problem that constructs a low-rank matrix (e.g. minimizing the nuclear norm: the sum of the singular values [57]; or matrix completion [58]). The approach used in our formulation is known as alternating minimization [39], which reconstructs the decomposed matrices at a fixed rank, pre-selecting an arbitrary low-rank value below the maximum potential rank of the system. Each row in X represents a separate voxel, each row in T represents a frame in time, and the rank is encoded through the columns of both matrices. An additional adaptation employed during prior generation is the forced orthogonalization of the system when alternating between the two subproblems where no alternate regularization exists (e.g. where λ_X_ = λ_T_ = λ_∇_ = 0).

Our reconstruction problem was solved using the minres.m function in MATLAB R2019a. NUFFT calculations used the Fessler toolbox [38]. Canonical correlations were calculated using the subspacea.m function [49] rather than the inbuilt canoncorr.m function, in order to avoid the extra alignment that occurs during demeaning (which is only significant for low canonical correlation scores). Reconstructions were run on a parallel computing cluster, using 4-core Intel CPUS (Ivybridge, Skylake, Haswell) ranging from 2.4-2.6 GHz with an 8GB maximum of RAM.

For windowing, a Tukey parameter of 0.4 was used with full-width half-maximum at $\frac{\pi*k\_max}{2R}$. For the generation of the priors, a 1D Tukey window was applied along each acquired blade in k-space, and a 2D version of the window was applied to the priors in Cartesian k-t space post prior-generation, but pre-final reconstruction with the full k-space. This ensured no leakage of energy into the higher frequencies, as the windowed data in a consistency term does not strictly enforce the output to only the central k-space.

The overall convergence criterion was a normalized cost function gradient; it was evaluated after the temporal subproblem in each cycle, relative to the cost function at the previous post-temporal iteration. The CCS metric was used to establish robustness within a given acceleration factor with respect to the convergence criterion, by reconstructing from different randomly initialized X and T matrices and measuring the agreement of those reconstructions with respect to the principal angles at different levels of convergence. Reconstructions were carried out across all k-t methods (except k-t PSF) on retrospective dataset A, and are shown in Supplementary Figure 1. R=52.36 was considered the maximum acceleration factor at which accurate comparisons could be made.

The differing size of the spatial and temporal subproblem means the spatial and temporal problems require different convergence and/or iteration parameters (typically there are 1-2 orders of magnitude more voxels than frames). We chose parameters that made the system spend 10x as long in the spatial subproblem (50 iterations per temporal subproblem, 500 per spatial subproblem, with a subproblem tolerance of 10^-15^ in case of early convergence). The effect of varying the number of iterations of each subproblem against the cycles between the subproblem is shown in Supplementary Figure 2. An internal iteration number of 50 was chosen to guarantee convergence, but this has the potential to be optimized for speed.

Toeplitz embedding exploits the Gram matrix (*E’E*) formed by Fourier encoding to produce a block Toeplitz structure. These can be embedded in block Circulant matrices, which can be fully explained by their first column, and are diagonalized by FFTs. Toeplitz Embedding speeds up the computation from O(N^2^) to O(NlogN). Mark Chiew’s tools for implementing can be found at <https://users.fmrib.ox.ac.uk/~mchiew/Tools.html>.


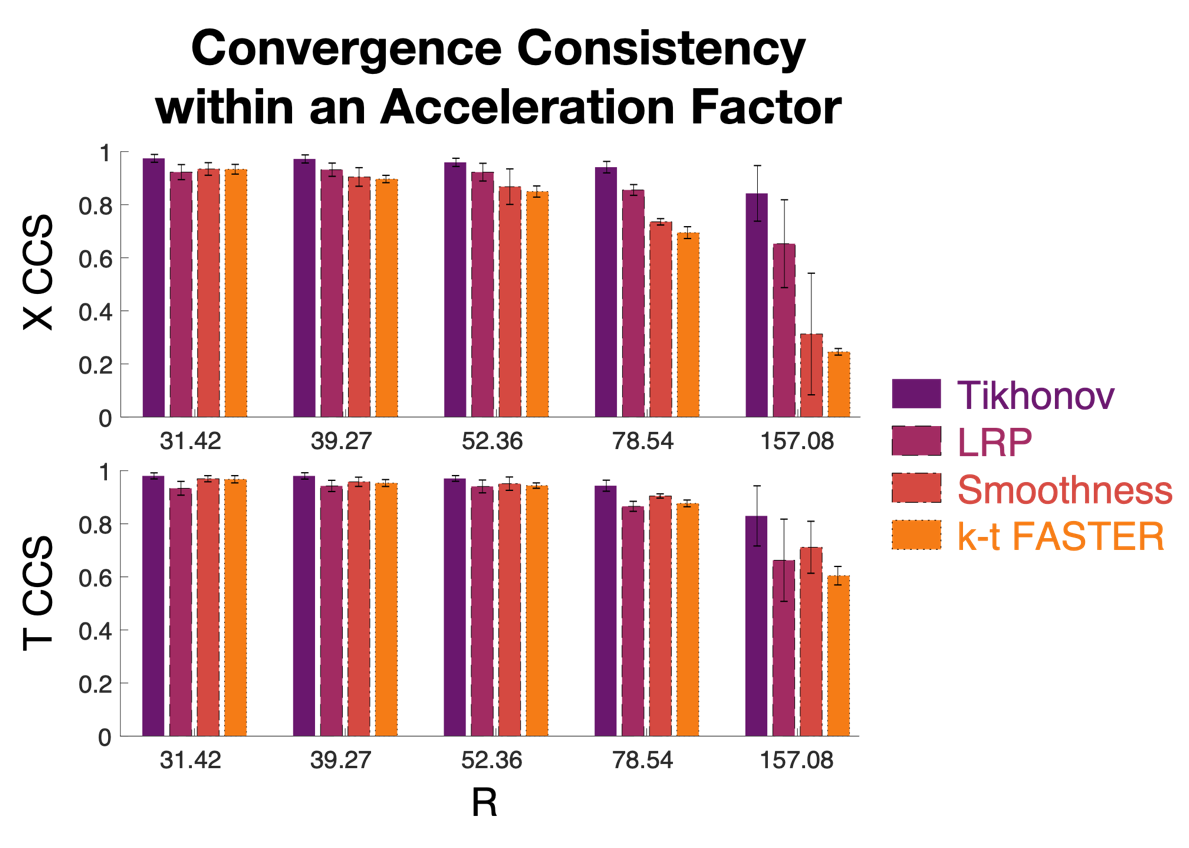
**Supplementary Figures – Retrospective Dataset A**


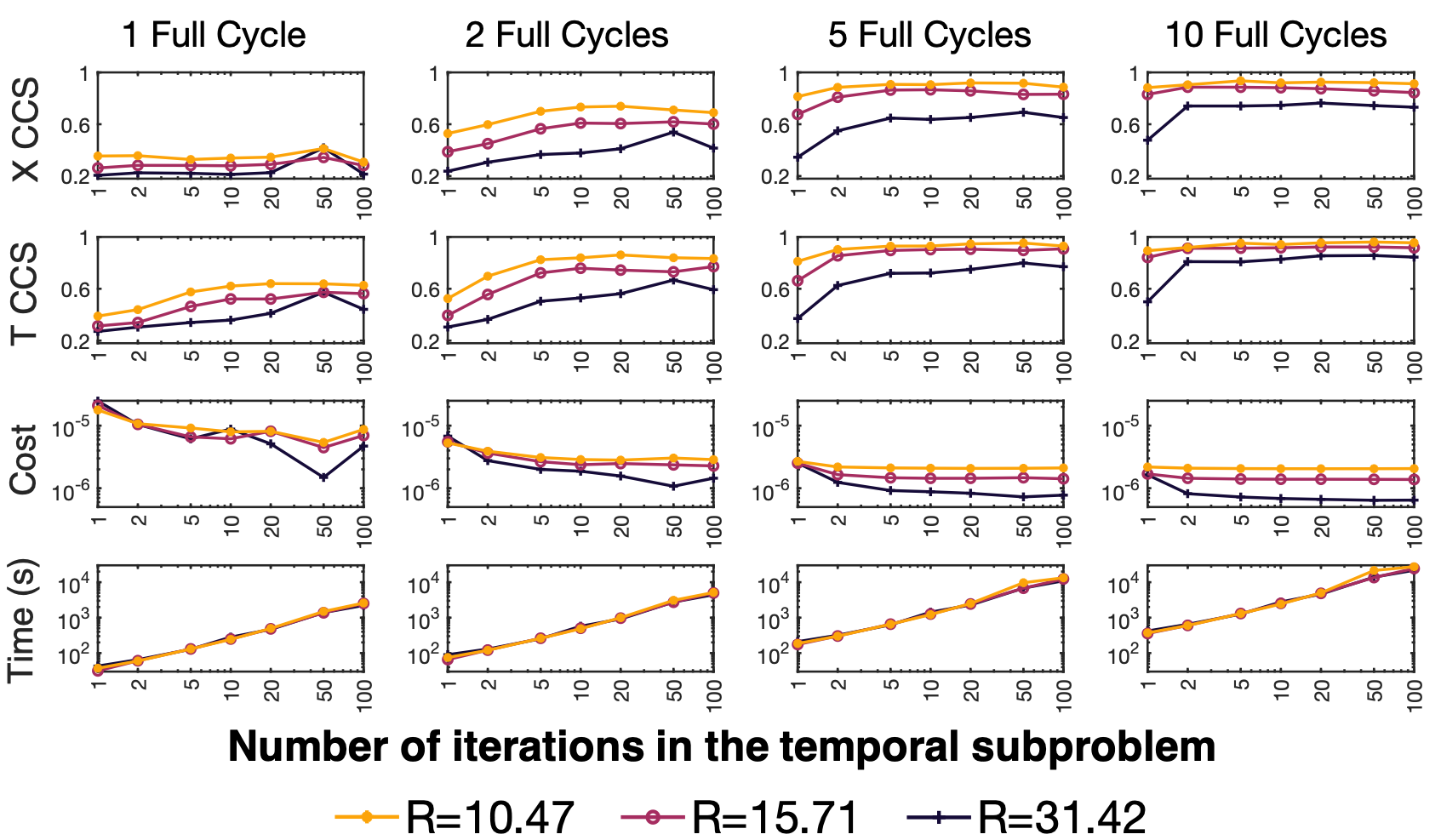
**Supplementary Figure 1:** A measure of the robustness of the different constraints in Retrospective Dataset A. At each different acceleration factor (columns), five different reconstructions with randomly orthogonal initialization and a temporal mean as the first component were carried out to a tolerance of ε≤10^-3^ (see Eq. 6), which was found to be representative of robustness. The mean CCS between these five different initializations is then shown in each bar, with one standard deviation shown as the range. It is worth noting that random non-orthogonal initializations showed much poorer convergence to a single solution, and that the necessity of k-t PSF initializing the temporal subspace with the temporal prior excluded it from this analysis. The robustness began to noticeably decrease at R>52.36 for all methods except Tikhonov, and the temporal subspace showed greater convergence consistency scores than the spatial subspace in nearly all cases.

**Supplementary Figure 2:** Various criteria (Row 1: X CCS, Row 2: T CCS, Row 3: Cost, Row 4: Time) are used to judge the reconstruction performance with varying iteration numbers in the subproblems. Three different acceleration factors are shown (R=31.42, 5 blades/frame; R=15.71, 10 blades/frame; R=10.47, 15 blades/frame) across a range of cycles (shown in each column). The number of iterations in the spatial subproblem was 10× higher. These results were acquired using alternating minimization k-t FASTER on Retrospective Dataset A.

**Supplementary Figures – Retrospective Dataset
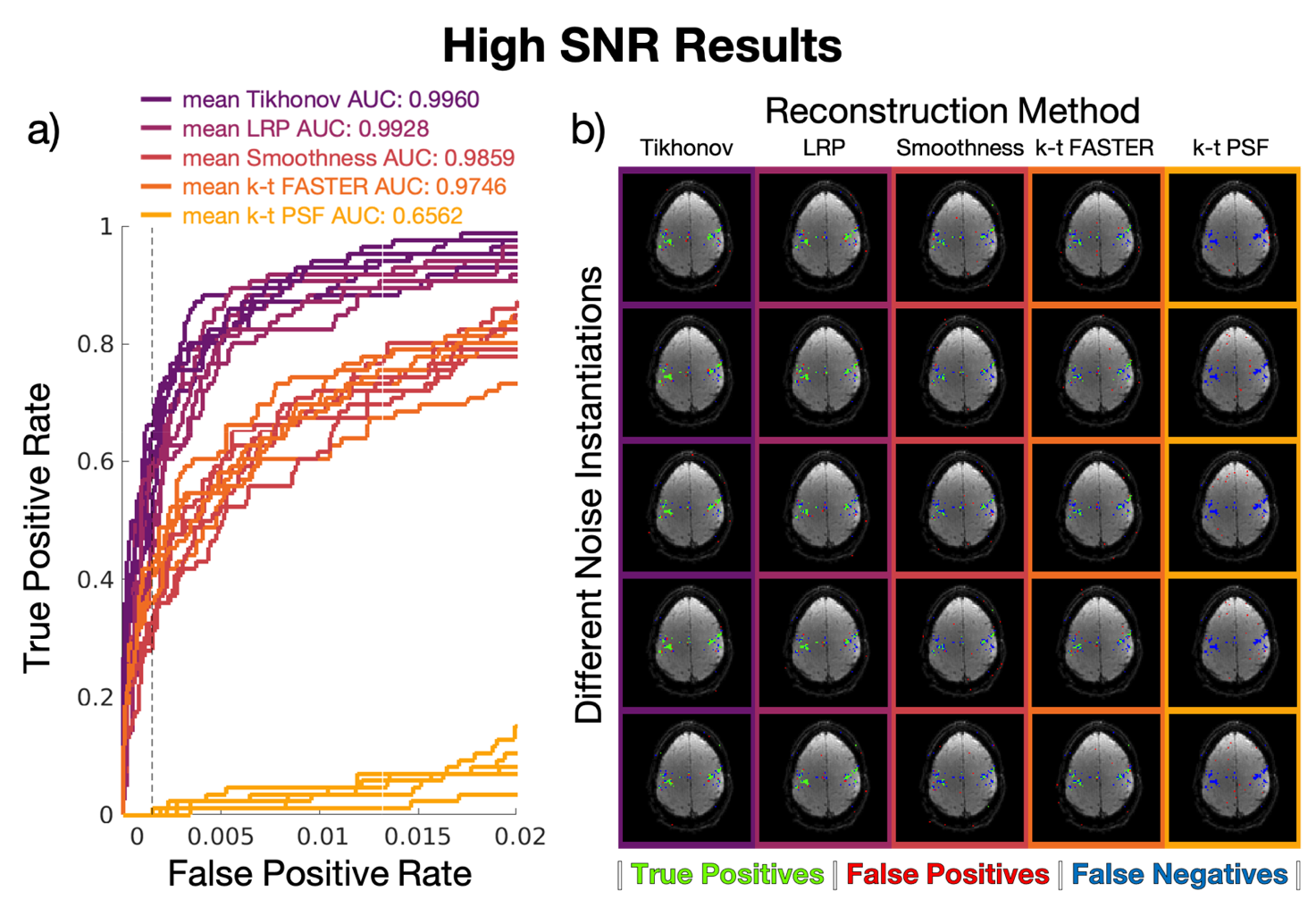
B**

**Supplementary figure 3:** The full set of reconstructions for high SNR in Retrospective Dataset B. a) The ROC curves for all five instantiations of the noise when subjected to the different reconstruction methods. The mean AUC across the entire curve is included in the legend. b) The activation maps of all methods for each instantiation of the noise.


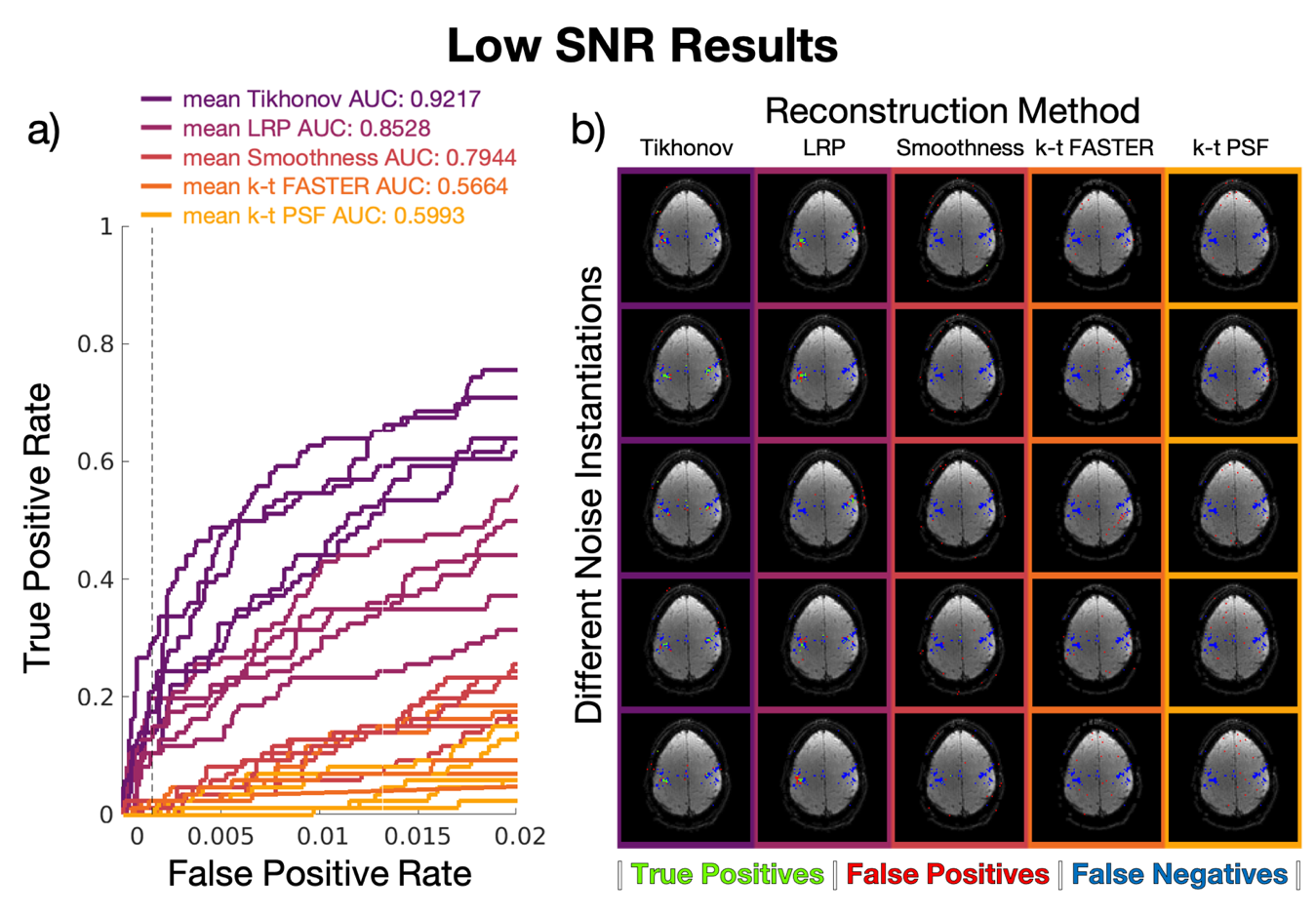

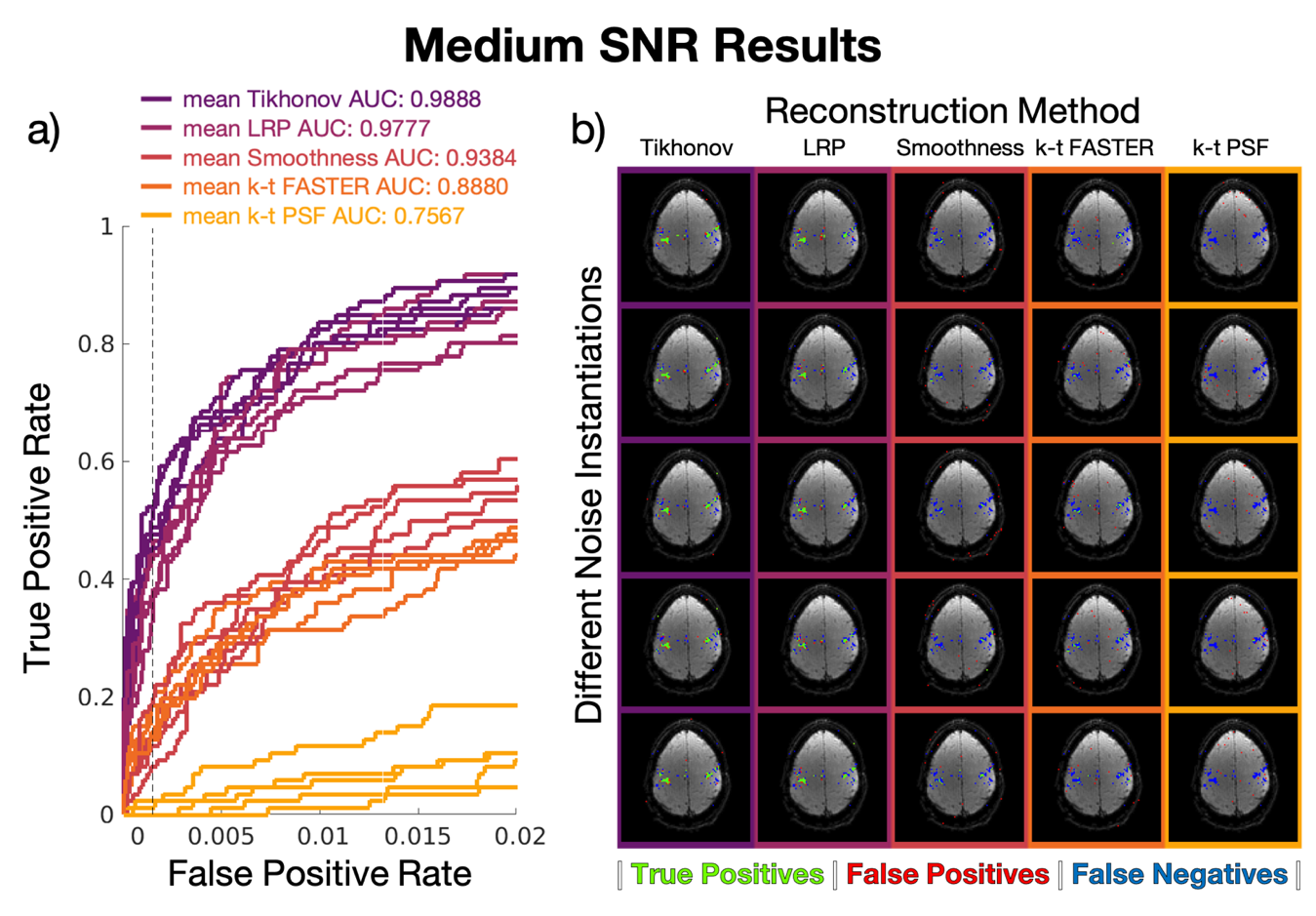
**Supplementary figure 4:** The full set of reconstructions for medium SNR in Retrospective Dataset B. a) The ROC curves for all five instantiations of the noise when subjected to the different reconstruction methods. The mean AUC across the entire curve is included in the legend. b) The activation maps of all methods for each instantiation of the noise.

**Supplementary figure 5:** The full set of reconstructions for low SNR in Retrospective Dataset B. a) The ROC curves for all five instantiations of the noise when subjected to the different reconstruction methods. The mean AUC across the entire curve is included in the legend. b) The activation maps of all methods for each instantiation of the noise.

*
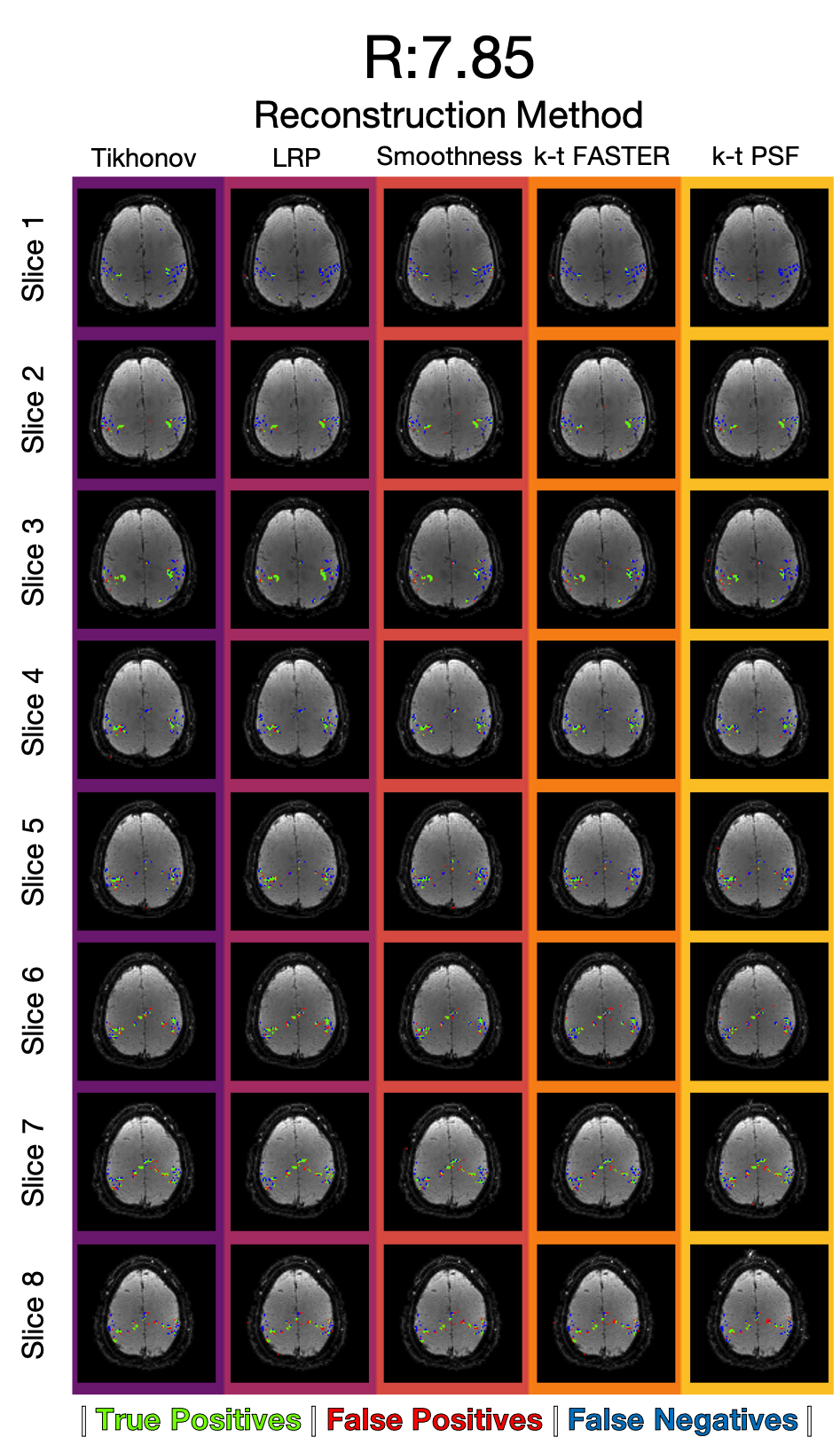
***Supplementary Figures – Prospective Dataset A**

**Supplementary figure 6:** The activation maps for all eight slices for Prospective Dataset A at R = 7.85 across the k-t reconstruction methods. The maps were thresholded according to the z-statistic equivalent to a false positive rate of 0.15% (Figure 8). Green pixels represent true positives, blue is false negatives, red is false positives.

*
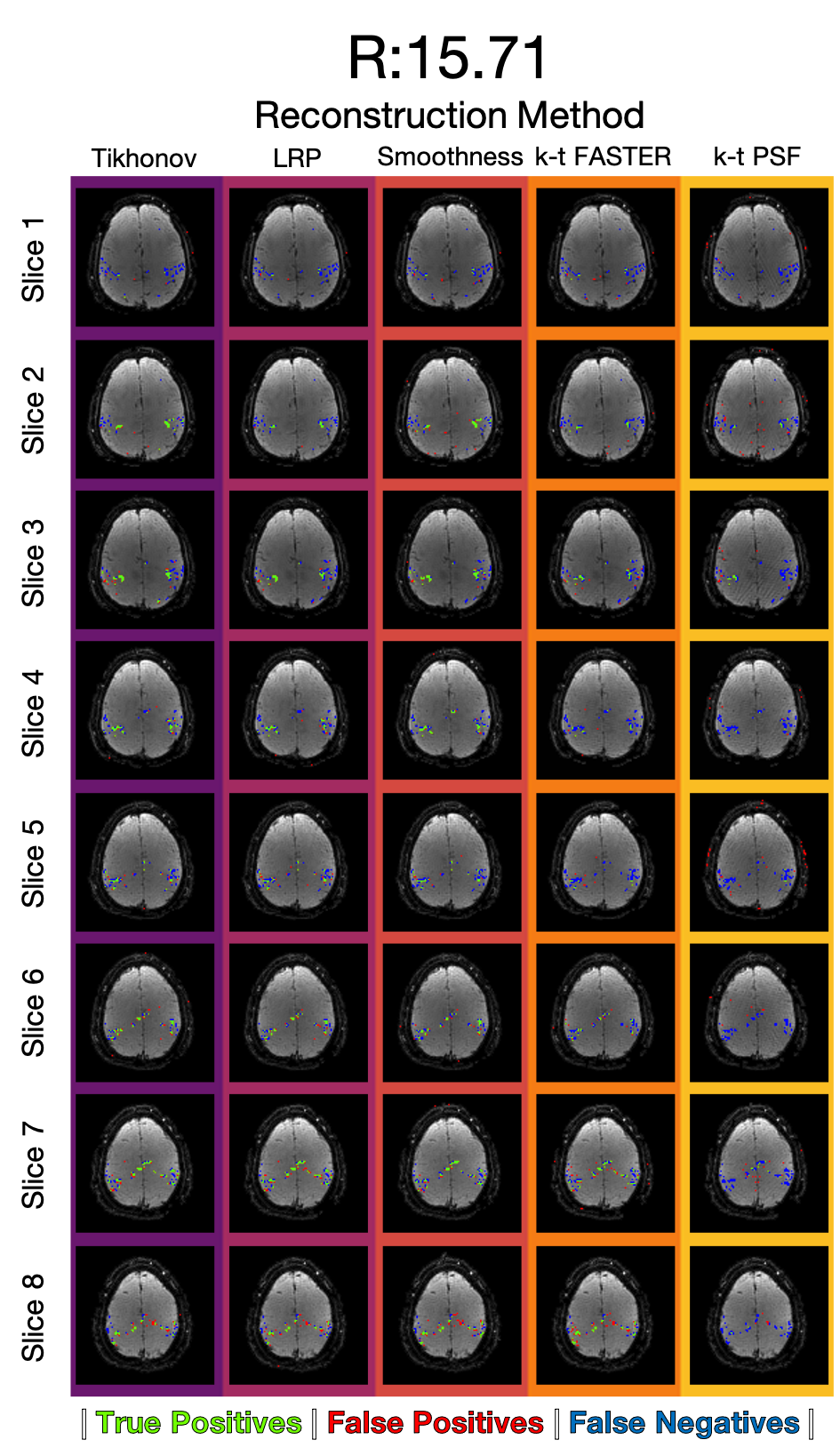
*

**Supplementary figure 7:** The activation maps for all eight slices for Prospective Dataset A at R = 15.71 across the k-t reconstruction methods. The maps were thresholded according to the z-statistic equivalent to a false positive rate of 0.15% (Figure 8). Green pixels represent true positives, blue is false negatives, red is false positives.

*
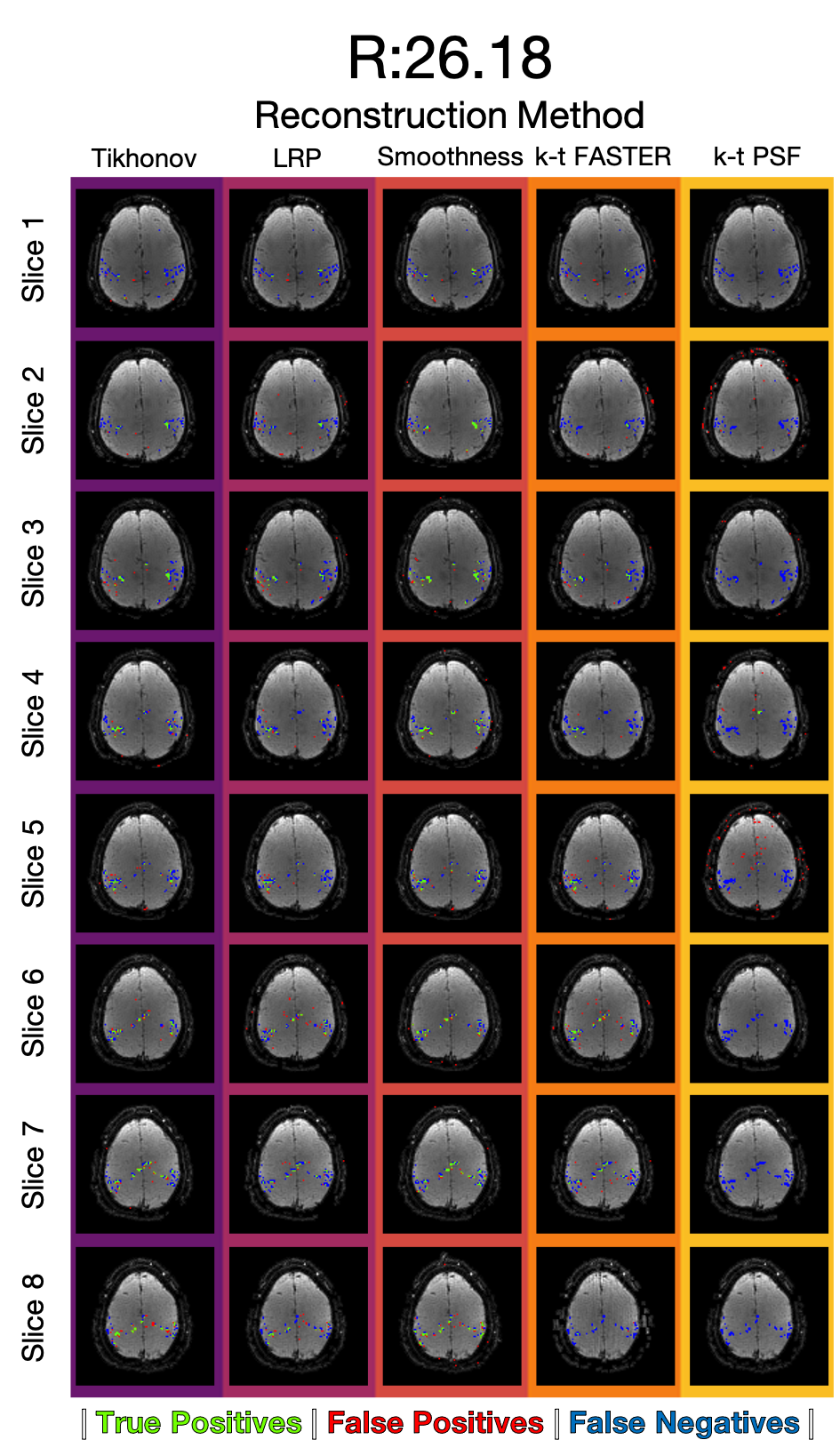
***Supplementary figure 8:** The activation maps for all eight slices for Prospective Dataset A at R = 26.18 across the k-t reconstruction methods. The maps were thresholded according to the z-statistic equivalent to a false positive rate of 0.15% (Figure 8). Green pixels represent true positives, blue is false negatives, red is false positives.

**
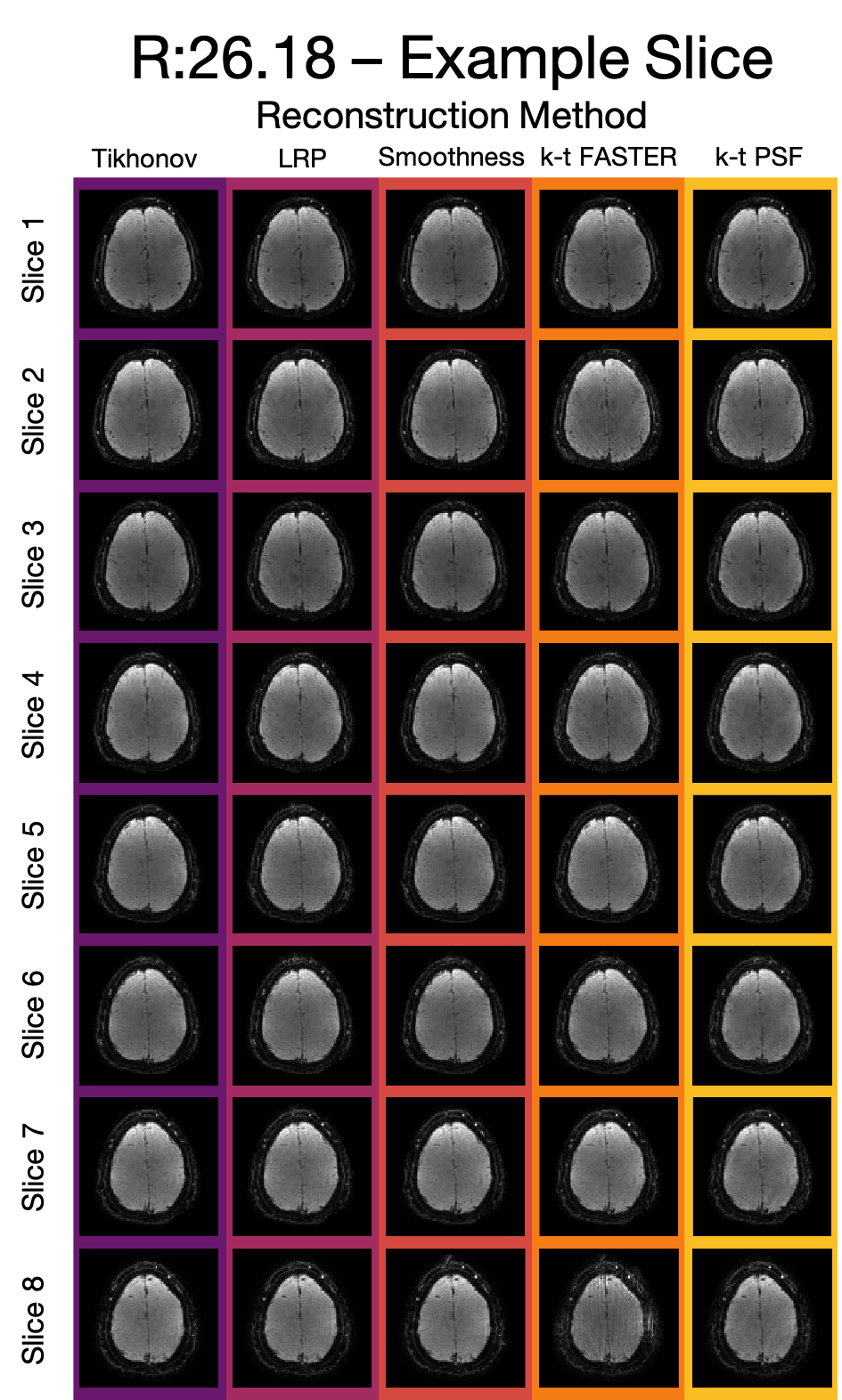
**

**Supplementary figure 9:** An example representation of the reconstruction from a single time frame of the reconstruction for all eight slices for Prospective Dataset A at R = 26.18 across the k-t reconstruction methods. All previous images show activation maps overlaid on the mean functional slice image.

**
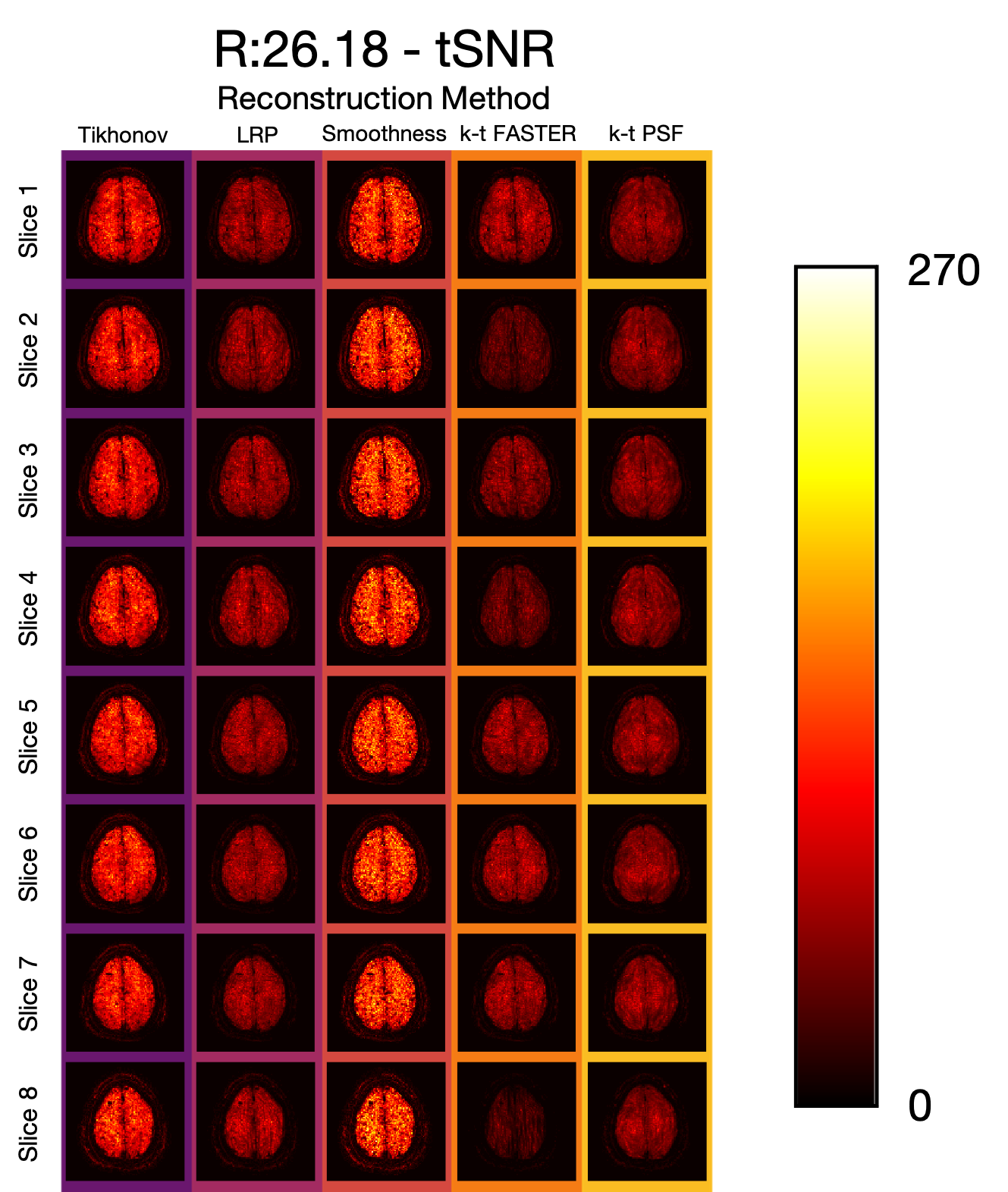
**

**Supplementary figure 10:** The tSNR calculated for all eight slices for Prospective Dataset A at R = 26.18 across the k-t reconstruction methods. tSNR is a more common metric for analyzing reconstruction quality. Under this metric, the Smoothness approach seems superior, with Tikhonov ahead of the remaining approaches. The tSNR of k-t FASTER appears to vary between slices, with this variation roughly equivalent to the quality of the z-statistic maps shown in Supplementary Figure 8.

**
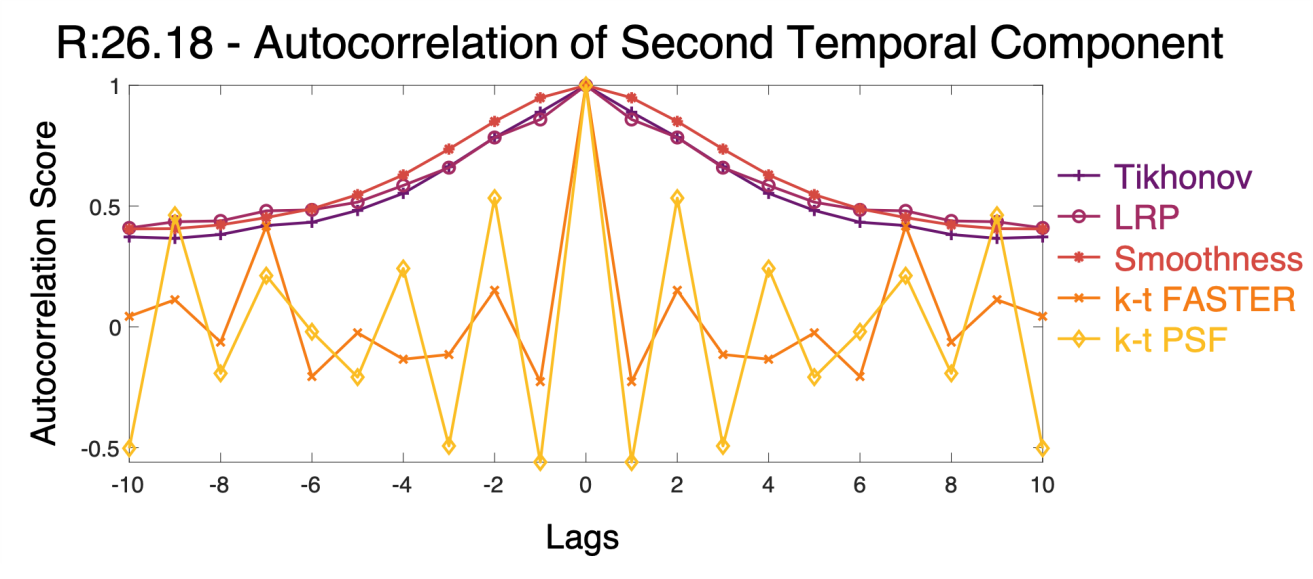
**

**Supplementary figure 11:** The autocorrelation of the second temporal component for Prospective Dataset A at R = 26.18 across the k-t reconstruction methods. The eight slices of each reconstruction were each combined into a 2D space-time matrix and then decomposed through an SVD. The first component represents largely static data (the unchanging brain). The second component represents a large proportion of the signal, with latter components becoming more noise-like. Although a wider autocorrelation is observed with the Smoothness constraint (and therefore fewer effective degrees of freedom), statistical comparisons did account for effective degrees of freedom via mixture modelling (as outlined in Section 2.2.3). The k-t FASTER and k-t PSF approaches did not produce high-fidelity reconstructions at this acceleration factor, and are more dominated by uncorrelated noise.
